# The impact of transposable elements on tomato diversity

**DOI:** 10.1038/s41467-020-17874-2

**Published:** 2020-08-13

**Authors:** Marisol Domínguez, Elise Dugas, Médine Benchouaia, Basile Leduque, José M Jiménez-Gómez, Vincent Colot, Leandro Quadrana

**Affiliations:** 1 Institut de Biologie de l’Ecole Normale Supérieure (IBENS), Centre National de la Recherche Scientifique (CNRS), Institut National de la Santé et de la Recherche Médicale (INSERM), Ecole Normale Supérieure, PSL Research University, 75005 Paris, France; 2 Genomic facility, Institut de Biologie de l’Ecole Normale Supérieure (IBENS), Département de biologie, École normale supérieure, CNRS, INSERM, Université PSL, 75005 Paris, France; 3 Institut Jean-Pierre Bourgin, INRAE, AgroParisTech, Université Paris-Saclay, 78000 Versailles, France

**Keywords:** Agricultural genetics, Genome-wide association studies, DNA transposable elements, Natural variation in plants

## Abstract

Tomatoes come in a multitude of shapes and flavors despite a narrow genetic pool. Here, we leverage whole-genome resequencing data available for 602 cultivated and wild accessions to determine the contribution of transposable elements (TEs) to tomato diversity. We identify 6,906 TE insertions polymorphisms (TIPs), which result from the mobilization of 337 distinct TE families. Most TIPs are low frequency variants and TIPs are disproportionately located within or adjacent to genes involved in environmental responses. In addition, genic TE insertions tend to have strong transcriptional effects and they can notably lead to the generation of multiple transcript isoforms. Using genome-wide association studies (GWAS), we identify at least 40 TIPs robustly associated with extreme variation in major agronomic traits or secondary metabolites and in most cases, no SNP tags the TE insertion allele. Collectively, these findings highlight the unique role of TE mobilization in tomato diversification, with important implications for breeding.

## Abstract (web summary)

Transposable element insertion polymorphisms (TIPs) are a potential source of large effect alleles. Here, the authors use genome resequencing data for 602 tomato accessions together with transcriptomic and extensive phenotypic information to investigate the contribution of TIPs to tomato diversity.

## Introduction

Tomatoes are the highest-value fruit and vegetable crop worldwide. Despite the recurrent genetic bottlenecks that have occurred since its domestication^1,2^, tomato exhibits extensive phenotypic variation, and the diversity we see today among cultivars is thought to result mainly from selection of rare alleles with large effects^3^. Nonetheless, while genomics-enabled genetics has revolutionized our ability to identify loci underlying domestication and improvement traits in virtually any crop^4–6^, our understanding of the genetic basis of crop diversity is still limited. This situation stems in part from the fact that, with few notable exceptions^7–11^, most genome-wide association studies (GWAS) consider only single-nucleotide polymorphisms (SNPs) and short indels^12,13^, when structural variants, which include gene presence/absence variants and typically segregate at low frequency, account for the largest amount of DNA sequence differences between individuals and cultivars^3,10,11,14^. Furthermore, the majority of structural variants result from the mobilization of transposable elements (TEs), which by themselves are potentially an important source of large-effect alleles^15^. Indeed, many TEs insert near or within genes^16^, and because of their epigenetic control as well as through the transcription factor-binding sites they harbor, TEs have the ability to alter gene expression and rewire gene expression networks^16,17^. Although numerous domestication and agronomic traits have been associated with particular TE insertions^15,18–22^, the specific contribution of TEs to the phenotypic diversification of crop species is still poorly documented. Here, we assess through a systematic analysis of 602 resequenced genomes the prevalence and impact of TE insertion polymorphisms (TIPs) among wild and cultivated tomatoes. We show that TIPs tend to have large transcriptional effects when located within or near genes and long-read Nanopore transcriptomics reveals that intronic TE insertions can generate multiple transcript isoforms with potential phenotypic consequences. Furthermore, GWAS detects numerous TIPs associated with variations in major agronomic traits or secondary metabolites. Importantly, these TIPs tend to affect loci that are distinct from those tagged by SNPs, illustrating the interest of incorporating TIPs into genomic-assisted breeding programs. Collectively, our approaches and findings provide a framework to study the implication of TIPs to crop diversity.

## Results

### Tomato mobilome composition

**Fig. 1:**
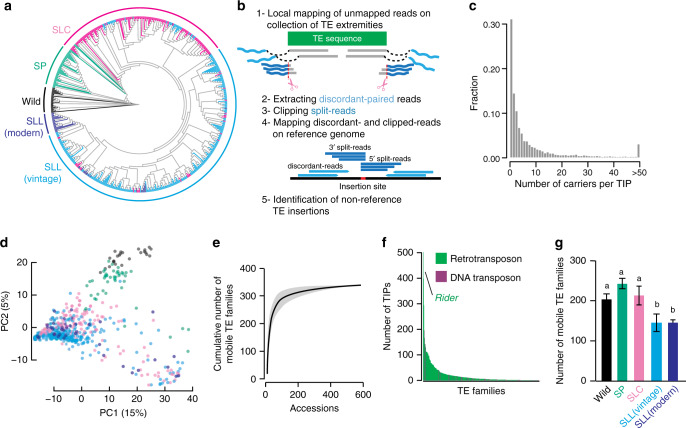
The tomato mobilome. **a** Phylogeny of the 602 tomato accessions analyzed, including wild tomato relatives (Wild), wild tomatoes (*S. pimpinellifolium*, SP), early domesticated tomatoes (*S. lycopersicum cerasiforme*, SLC), and cultivated tomatoes (*S. lycopersicum lycopersicum* vintage and modern, SLL). **b** Schematic representation of the SPLITREADER bioinformatics pipeline used to identify TE insertion polymorphisms (TIPs) using split- and discordant reads. **c** Distribution frequency of allele counts for TIPs. **d** Principal component analysis based on TIPs. Colors represent tomato groups as indicated in (**a**). **e** Cumulative plot of the number of mobile TE families detected with increasing numbers of accessions. Shaded bands represent ±95% CI. **f** Number of detected TIPs per TE family. **g** Number of mobile TE families detected in each tomato group. Data are mean ±95% CI obtained by 100 bootstraps, and statistical significance for differences were obtained by a randomization test. Source data of Fig. 1a, f are provided as a Source Data file.

The tomato reference genome (*Solanum lycopersocum* cv. Heinz 1706, release SL2.5) contains 665,122 annotated TE sequences belonging to 818 families^23^. The vast majority of these annotations correspond to ancestral TE copies that have degenerated to different degrees and potentially lost their ability to transpose^24^. To investigate the composition of the tomato mobilome, i.e., the set of TE families with recent mobilization activity, we analyzed short-read whole-genome resequencing data available for 602 tomato accessions^2,25,26^. This dataset contains wild tomato relatives (Wild, Fig. 1a) and spans the Lycopersicon clade, which regroups wild tomatoes (*S. pimpinellifolium*, SP), early domesticated tomatoes (*S. lycopersicum cerasiforme*, SLC), and cultivated tomatoes (*S. lycopersicum lycopersicum* vintage and modern, SLL). To detect additional, i.e., non-reference, TE insertions in each genome sequence, we deployed a refined version of the SPLITREADER pipeline^27^ (Fig. 1b, see “Methods”). We restricted our analysis to the 467 TE families with annotated copies longer than 1 kb in the reference genome. These families represent the full range of Class I LTR and non-LTR retroelements (i.e., *GYPSY*, *COPIA*, and *LINE* superfamilies) and Class II DNA transposons (i.e., *MuDR*, *hAT*, and *CACTA* superfamilies), which move through copy-and-paste and cut-and-paste mechanisms, respectively. After filtering low-quality calls (see “Methods”), 6906 non-reference TE insertions remained for downstream analysis (Supplementary Data 1). Most TE insertions were present in one or a few tomato accessions only (Fig. 1c), suggesting that they occurred recently. Nonetheless, cluster analysis based on these 6906 TIPs recapitulated the phylogenetic relationship between accessions previously determined using SNPs (Fig. 1d)^2,3^.

TIPs were contributed by 337 TE families in total, which likely represent the near-complete composition of the tomato mobilome. Indeed, most TE families with TIPs could be detected using only ~200 of the 602 resequenced genomes (Fig. 1e), and the majority (84%) of TIPs resulted from the mobilization of *GYPSY* and *COPIA* LTR retrotransposons (Fig. 1f; Supplementary Fig. 1a). The *COPIA RIDER* family, which generated insertion mutations with important agronomic implications^21,22,28,29^, contributes the highest number (507) of TIPs overall. Mobilome composition varies substantially among tomato groups and, as expected, is the richest in the genetically diverse SP group (~230 TE families, Fig. 1g). However, despite the loss of genetic diversity associated with domestication (Supplementary Fig. 1b)^2,3^, the mobilome composition of early domesticated SLC is only marginally reduced compared with that of SP (210 vs. 230 TE families, Fig. 1g). This last observation is consistent with the recurrent hybridization between SLC and SP^1^, and the unique ability of TEs to invade new genomes^30^. In contrast, vintage and modern SLL have a more reduced mobile composition (~150 TE families, Fig. 1g), in keeping with the strong genetic bottleneck caused by the post-Columbian introduction of tomato to Europe.

### TIP landscape and transcriptional impact

**Fig. 2:**
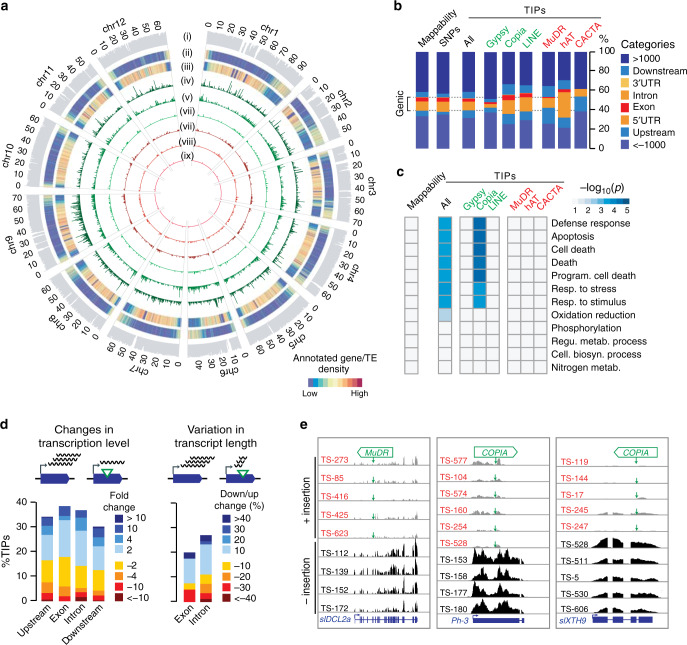
Landscape and transcriptional impact of TIPs. **a** Chromosomal short-read mappability (i) and distributions of reference genes (ii) and TEs (iii) as well as TIPs by superfamily (iv–ix) across the 12 chromosomes of tomato genome. (iv) *GYPSY*, (v) *COPIA*, (vi) *LINE*, (vii) *MuDR*, (viii) *hAT*, and (ix) *CACTA*. **b** Distribution of TIPs over genic features. UTR, untranslated transcribed region. **c** GO-term analysis of genes with TIPs. **d** Proportion of TIP-containing genes with changes in transcription level or variation in transcript length in relation to the presence/absence of the TE insertion. **e** Genome browser view of RNA-seq coverage for three TIP-containing genes in accessions carrying or not the TE insertions. Green arrows indicate the position of TE insertion sites. Source data of Fig. 2c, d are provided as a Source Data file.

Whereas TE sequences present in the reference genome are enriched in pericentromeric regions^23^, TIPs are distributed more equally along chromosomes (Fig. 2a). Nonetheless, superfamily-specific integration patterns are evident. For instance, TIPs corresponding to *COPIA* and many other TE superfamilies are found preferentially within or near genes, while *GYPSY* TIPs cluster in pericentromeric regions (Fig. 2a, b). Importantly, genes harboring TIPs are overrepresented in functions related to response to pathogens or other environmental stresses (Fig. 2c). This overrepresentation is driven by *COPIA* insertions and likely reflects integration preferences rather than relaxed purifying selection or detection biases, which should affect all types of TIPs. Indeed, experimental evidence indicates that *COPIA* integrates preferentially within environmentally responsive genes in *Arabidopsis* and rice^31^.

In many organisms, including plants and animals, TIPs have been associated with large transcriptomic changes^14,27,32–34^. To assess the impact of TIPs on gene expression in tomato, we used RNA-seq data obtained from breaker fruits for 400 accessions^35^. We considered all genes harboring a TIP within 1 kb, and compared transcript levels between accessions carrying or lacking the insertion. TIPs associated with two-fold or more changes in gene expression are proportionally more frequent when located in exons and introns (43% and 37%, respectively) than in other gene compartments (Fig. 2d). Furthermore, changes are either positive or negative, consistent with the notion that TE insertions can affect gene expression in multiple ways. To explore further these transcriptional effects, we compared RNA-seq coverage upstream and downstream of insertion sites (Fig. 2d). This analysis uncovered additional TIPs affecting gene expression, and revealed that between 20% and 28% of genic TIPs interfere with transcript elongation when exonic or intronic, respectively. Taken together, these results indicate that TIPs residing within the transcribed part of genes have pervasive and complex effects.

Consistent with the observed overrepresentation of TIPs within specific gene ontology categories, expression of immune- and stress-responsive genes was particularly affected by TIPs (Fig. 2e). For instance, we uncovered a rare *MuDR*-containing allele of the gene *slDCL2a* (*Solyc06g048960*), which is involved in resistance against RNA viruses^36^. As the intronic insertion is associated with a severe reduction in transcript level, accessions carrying the rare allele could be more susceptible to viral attacks. Likewise, an exonic *COPIA* insertion within the CC-NB-LRR gene *Ph-3* (*Solyc09g092310*), which confers broad resistance to *Phytophthora infestans*^37,38^, is associated with transcript truncation and could therefore cause increased susceptibility to this pathogen. We also identified TE insertions with potential beneficial effects. For example, the exonic *COPIA* insertion in the gene *slXTH9* (*Solyc12g011030)*, which encodes a xyloglucan endotransglucosylase/hydrolase preferentially expressed during fruit ripening^39^, is associated with a near-complete loss of expression. Given the key role of *slXTH9* in fruit softening^24^, this natural loss-of-function allele could potentially be harnessed to breed tomato fruits with harder texture and longer shelf life^40^.

### TIPs as an unregistered source of phenotypic variants

**Fig. 3:**
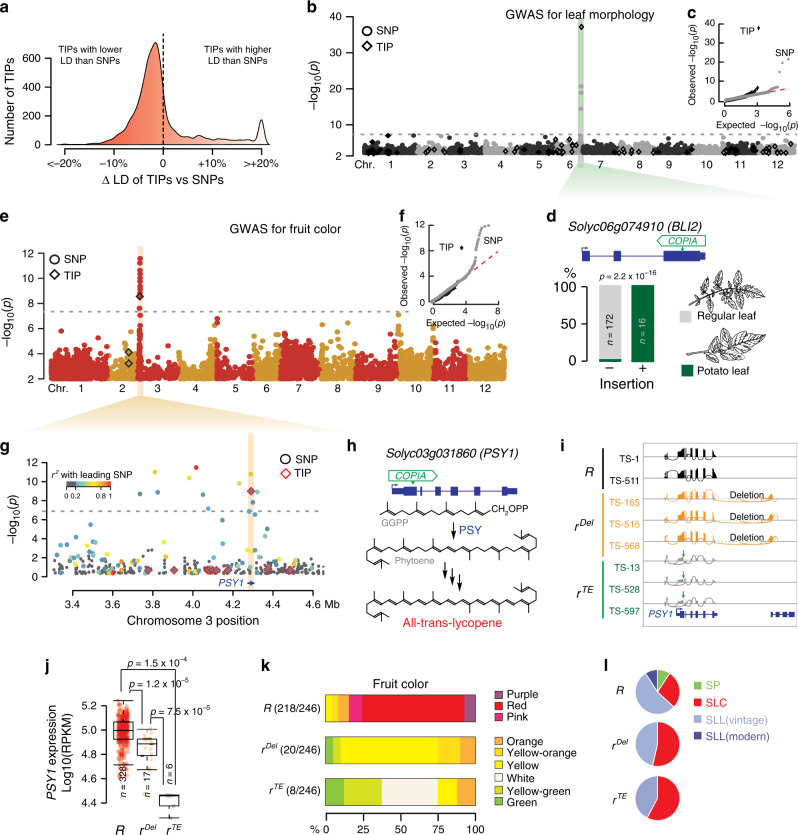
TIPs as an unregistered source of phenotypic variants. **a** Distribution of the proportion of SNPs that are in lower or higher linkage disequilibrium (LD) with TIPs or other SNPs. **b** Manhattan plot of SNP- and TIP-based GWAS (circles and diamonds, respectively) for leaf morphology. **c** Observed and expected distribution of *p* values for SNP- and TIP-GWAS (gray circles and black diamonds, respectively). **d** Leaf morphology of accessions carrying or lacking a *COPIA* insertion within *BLI2*. Statistical significance for differences was obtained using two-sided Fisher test. **e** Manhattan plot of SNP- and TIP-based GWAS (circles and diamonds, respectively) for fruit color. **f** Observed and expected distribution of *p* values for SNP- and TIP-GWAS (gray circles and black diamonds, respectively). **g** Manhattan plot of SNP- and TIP-based GWAS (circles and diamonds, respectively) around *PSY1*. Colors indicate the linkage disequilibrium (*r*^2^) with the leading variant. **h** Structure of the *PSY1* gene with the position of the *RIDER* insertion and simplified representation of lycopene biosynthesis. **i** Genome browser view of RNA-seq coverage over *PSY1* for accessions carrying the wild-type (*R*) or mutant alleles (*r*^*del*^ and *r*^*TE*^) for the gene. **j** Quantification of *PSY1* expression. For each boxplot, the lower and upper bounds of the box indicate the first (Q1) and third (Q3) quartiles, respectively, the center line indicates the median, and the whiskers represent data range, bounded to 1.5 * (Q3–Q1). Statistical significance for differences (not adjusted for multiple comparisons) was obtained using a two-sided MWU test. **k** Fruit color of accessions with the distinct alleles of *PSY1*. **l** Distribution of the three *PSY1* alleles between tomato groups. GGPP geranylgeranyl diphosphate. Source data of Fig. 3d, e, j are provided as a Source Data file.

To assess more systematically whether TIPs are a potentially important source of phenotypic variation, we first measured the proportion of TIPs in high-linkage disequilibrium (LD, *r*^2^ > 0.4) with SNPs. This proportion was much lower than for SNPs in high LD with other SNPs (Fig. 3a). This result was confirmed using a set of 56 visually validated TIPs (Supplementary Fig. 2a), indicating that the lower LD observed for TIPs compared with SNPs cannot be fully explained by reduced sensitivity and specificity of TIP detection. Conversely, and in agreement with previous findings in Arabidopsis^33^, maize^11^, grapevine^10^, and humans^14^, rare TIPs (MAF < 1%) tend to have lower LD with nearby SNPs than more common TIPs (Supplementary Fig. 2b, c). In addition, most TIPs in high LD with SNPs are located on chromosome 9 (Supplementary Fig. 2d), consistent with modern tomatoes harboring on that chromosome a large introgressed segment from wild tomatoes^2^. Based on these observations and because TE insertions tend to generate large-effect alleles, we reasoned that even when low frequency variants, TIPs could still be used for TIP-GWAS^9^. We considered TIPs with MAF > 1% and with less than 20% of missing data in GWAS for 17 important agronomic traits in tomato, including determinate or indeterminate growth, simple or compound inflorescences, leaf morphology, as well as fruit color, shape, and taste. Importantly, given the reduced sensitivity and specificity of TIP detection, which can increase the probability of finding false associations, we curated all putatively associated TIPs by visual inspection. These TIP-GWAS uncovered a total of nine high-confidence loci associated with five traits, including fruit color and leaf morphology (Supplementary Fig. 3a). These two traits were previously linked to TE insertions^28,41^, thus validating our TIP-GWAS approach. Moreover, association with leaf morphology is much stronger for the TIP than for any SNP (Fig. 3b–d), suggesting that TIP-GWAS was able to pinpoint the causal variant. In addition, most TIP associations could not be identified using SNPs (Supplementary Fig. 3b), demonstrating the interest of considering TIPs in addition to SNPs in GWAS. For instance, our TIP-GWAS revealed a strong association between a *RIDER* insertion within the gene *PSY1*, which encodes a fruit-specific phytoene synthase, and yellow fruit (Fig. 3e–h). Incidentally, our SNP-GWAS revealed another variant of *PSY1* associated with yellow fruit (Fig. 3g). Local assembly using short reads indicated that this alternative allele, which we named *r*^Del^ to distinguish it from the previously identified *r*^TE^ allele, contains an ~6-kb deletion that bridges the last exon of *PSY1* with the next gene (*Solyc03g031870*) downstream (Fig. 3i). Together, *r*^TE^ and *r*^Del^ account for 60% of yellow tomato accessions, and those carrying the *r*^TE^ allele display lower expression levels of *PSY1* and yellower fruit than accessions with the *r*^Del^ allele (Fig. 3j, k). Moreover, we detected the *r*^TE^ and *r*^Del^ alleles in several SLC and SLL vintage accessions but in none of the wild tomatoes (*S. pimpinellifolium*) and wild relatives (Fig. 3l), which suggests that *r*^TE^ and *r*^Del^ arose after domestication. Also, while the *RIDER* insertion affected a common haplotype of *PSY1* shared among early domesticated and improved tomatoes, the ~6-kb deletion affected a rare haplotype containing numerous SP-derived sequences (Supplementary Fig. 4). Together, these results suggest that the first tomato cultivar introduced in Europe during the sixteenth century, which was reported to be yellow^42^, harbored the *r*^TE^ allele.

**Fig. 4:**
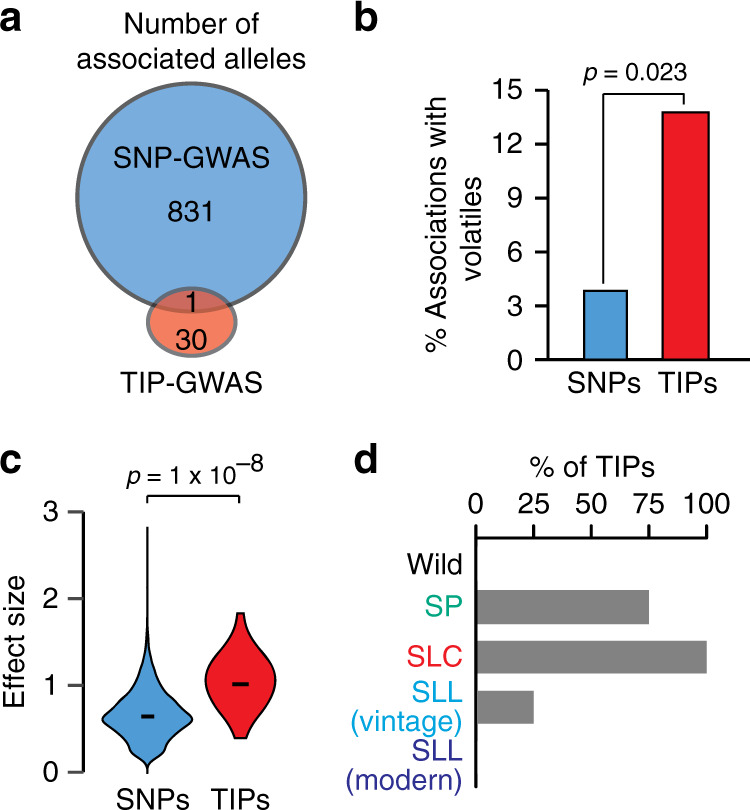
TIP associations with secondary metabolism. **a** Significant associations detected by SNP- and TIP-GWAS and their overlap. **b** Percentage of identified loci associated with variation in volatiles. Statistical significance for differences was obtained using a two-sided Fisher test. **c** Effect size for association signals detected in SNP- and TIP-GWAS. Statistical significance for differences was obtained using a two-sided MWU test. **d** Percentage of TIPs with significant associations present within each of the five tomato groups. Source data of Fig. 4a, b, d are provided as a Source Data file.

To investigate further the specific contribution of TIPs to trait variation in tomato, we conducted SNP- and TIP-GWAS on 1012 metabolic phenotypes measured for more than 397 accessions^25,35^. In total, 846 and 41 associations with 369 and 30 metabolites were identified by SNP- and TIP-GWAS, respectively (Fig. 4a). Of the 41 associations, 31 were confirmed by visual inspection of the underlying TIPs and were considered further. Remarkably, except in one case, the TE-containing allele is not tagged by any SNP, and 14 TIPs affect loci not identified by SNP-GWAS. Moreover, TIPs unlike SNPs are predominantly associated with variation in volatiles (Fig. 4b), a class of secondary metabolites that are implicated in defense response and interaction with other organisms^43^. This skewing of TIP associations is readily explained if one considers that constraints are lower on secondary than on primary metabolism, and that on average, the effect size of TIPs is much larger than that of SNPs (Fig. 4c). Finally, because almost all of the TE-containing alleles detected using our TIP-GWAS are present in SLC accessions (Fig. 4d), their contribution to phenotypic diversification is higher among early domesticated tomatoes.

### A key TIP for tomato flavor

**Fig. 5:**
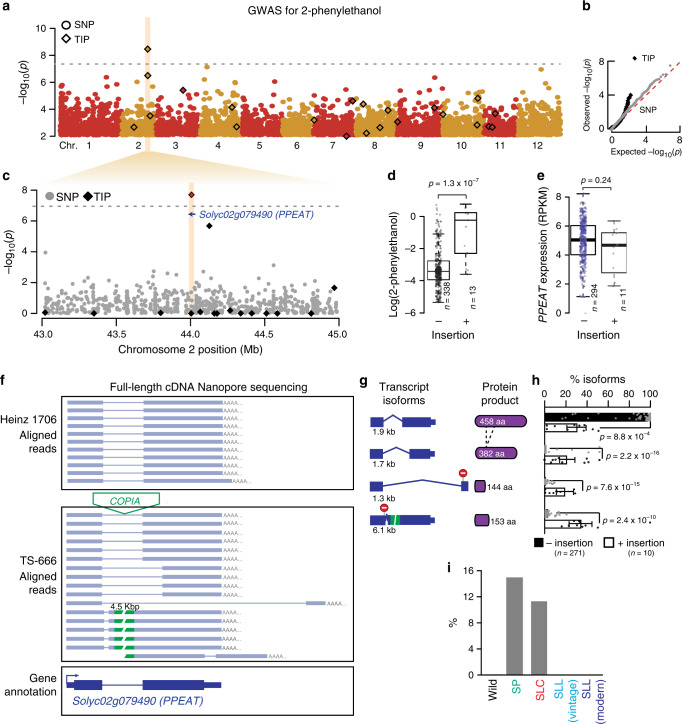
A key TIP for tomato flavor. **a** Manhattan plot of SNP- and TIP-based GWAS (circles and diamonds, respectively) for 2-phenylethanol. **b** qq-plot depicting observed and expected distribution of *p* values for SNP- and TIP-GWAS (gray circles and black diamonds, respectively). **c** Detailed view of the Manhattan plot for 2-phenylethanol spanning *Solyc02g079490 (PPEAT)*. **d**. 2-phenylethanol levels in accessions carrying or not the intronic *COPIA* insertion. Statistical significance for differences was obtained using one-sided t test. **e**. *PPEAT* expression level in accessions carrying or not the intronic *COPIA* insertion. Statistical significance for differences was obtained using two-sided MWU test. **f** Genome Browser view of full-length cDNA nanopore reads from accessions carrying or not the intronic *COPIA* insertion. **g** *PPEAT* transcript isoforms, protein products, and abundance of transcript isoforms in accessions carrying or not the associated TE insertion. Data are mean ± s.d., and statistical significance for differences was obtained using two-sided MWU test. **h** Frequency (%) of the intronic *COPIA*-containing allele in each of the five tomato groups. PPEAT putative 2-phenylethanol acyl transferase. For each boxplot, the lower and upper bounds of the box indicate the first (Q1) and third (Q3) quartiles, respectively, the center line indicates the median, and the whiskers represent data range, bounded to 1.5 * (Q3–Q1). Source data of Fig. 5d, e, g, h are provided as a Source Data file.

Our TIP-GWAS revealed a *COPIA* LTR-retrotransposon insertion that is absent in modern cultivars and which is associated with high levels of 2-phenylethanol (Fig. 5a–d), a volatile that gives a pleasant flowery aroma to heirloom tomatoes^44^. This TE insertion is located in the single intron of gene *Solyc02g079490*, which is preferentially expressed in ripe fruits and encodes a protein with high similarity (63% aa identify) with a cinnamyl alcohol Acyl-CoA transferase^45^ (Supplementary Fig. 5a, b). Consistent with a potential role of *Solyc02g079490* in the accumulation of 2-phenylethanol, the introgression line (IL) 2.3^46^, which harbors the lowly expressed *S. pennellii* allele of *Solyc02g079490*^47^, also accumulates more 2-phenylethanol compared with the modern cultivar M82 (Supplementary Fig. 5c)^48^. Thus, *Solyc02g079490* likely encodes a putative 2-phenylethanol Acyl-CoA transferase (PPEAT) involved in the esterification of 2-phenylethanol, which otherwise accumulates in fruits.

Although the intronic *COPIA* insertion does not appear to affect the expression levels of *Solyc02g079490* (Fig. 5e), hereafter referred to as *PPEAT*, we noted numerous transcript isoforms in accessions carrying the insertion compared to a single predominant transcript otherwise (Supplementary Fig. 5d). To characterize these additional transcripts further, we performed full-length cDNA Nanopore sequencing of ripe fruit samples from two accessions, one carrying and one lacking the intronic *COPIA* insertion. We uncovered in this manner at least three additional transcript isoforms, all of which result from alternative splicing (Fig. 5f). Moreover, the *COPIA*-containing intron (>5 kb), which is one of the largest intron genome-wide based on our Nanopore sequencing (Supplementary Fig. 5e), is spliced out in most cases. Nonetheless, this large intron is retained in one isoform, thus leading to an unusually long transcript (Fig. 5g; Supplementary Fig. 5e). All of the alternative isoforms incorporate premature stop codons or encode proteins that lack highly conserved catalytic domains (Fig. 5g; Supplementary Fig. 5f). Based on these findings, we reanalyzed the RNA-seq data obtained for 400 accessions. This reanalysis confirmed that truncated isoforms are almost exclusively associated with the intronic *COPIA* insertion, and revealed that they make up around 60% of all *PPEAT* transcripts (Fig. 5h). Based on these additional findings, we propose that the *COPIA* insertion generated a hypomorphic *PPEAT* allele, which would explain the overaccumulation of 2-phenylethanol. This could be formally demonstrated in the future by removing the *COPIA* insertion through genome editing. Also, because the insertion is absent from wild relatives, but present at intermediate frequency in wild (*S. pimpinellifolium*) and SLC tomatoes (Fig. 5i), we speculate that the *COPIA*-containing allele of *PPEAT* predated domestication, and that it was selected in early domesticated tomatoes but not in modern varieties, which are notorious for their poor flavor^44^.

## Discussion

Cultivated tomato has a complex history of domestication and improvement, characterized by two successive genetic bottlenecks, followed since modern breeding by several introgression events from wild tomatoes and relatives to replenish the limited pool of disease-resistance genes^1,10,49^. Despite a relatively narrow genetic diversity, the more than 25,000 cultivars grown around the world today exhibit an extraordinary phenotypic diversity, and the underlying allelic variants are being progressively identified, thanks to the advent of high-throughput genome sequencing. Here, we show that TIPs, which to date have been ignored from population genomic studies in tomato, are an important diversifying force to consider, as has been proposed for other plant species^27,33,34,50–55^. For instance, GWAS in rice for grain length and width using respectively structural variants and TIPs uncovered associations that could not be detected using SNP data^8,9^. Moreover, in the case of grain width, the associated TIP is very rare and in low LD with nearby SNPs. Likewise, we found that most TE insertions in tomato are low-frequency variants rarely tagged by SNPs. Thus, our findings reinforce the notions that TIPs and SNPs contribute distinct phenotypic variants, and that TIPs identified in GWAS as leading variants are likely causal^7–10^, which opens up the way for their use for future breeding.

We based our study on TIPs using 467 TE families that represent most of the retrotransposons and DNA transposon families present in the reference tomato genome. However, we did not consider small-length TE families, such as SINEs and MITEs, which are also likely to contribute to phenotypic diversity^53^, but that are difficult to analyze using short reads because of their small size and very high copy number in many genomes. The implementation of long-read sequencing should remedy this problem, as well as that of the low sensitivity and specificity of TIP detection using short-read sequencing technologies. Indeed, a landmark analysis of 100 tomato genomes based on long-read Nanopore sequencing revealed thousands of structural variants, mostly involving TEs that intersect genes and *cis*-regulatory regions^56^. This and other studies using long-read sequencing^10,11,57^ suggest that we will soon be in a position to assess comprehensively the contribution of TE insertions, as well as of the structural variants they can generate through recombination or other means, to crop diversity.

The composition of the tomato mobilome, as defined here, appears to be substantially reduced following the post-Columbian introduction of tomato to Europe. This observation may reflect the strong genetic bottleneck this introduction created, as well as the increased levels of inbreeding that ensued, as the latter favors the accumulation of deleterious mutations, and is therefore expected to compromise the long-term survival of accessions with high mobilome activity^15^. Whether introgression from wild germplasms used in modern breeding can alter this picture by enabling new TE mobilization remains to be determined.

## Methods

### Detection of TIPs

Illumina resequencing data from 602 tomato accessions were downloaded from EBI-ENA and aligned to the tomato genome reference (version SL2.5) using Bowtie2 v.2.3.2 (arguments –mp 13 –rdg 8,5 –rfg 8,5 –very-sensitive), and PCR duplicates were removed using Picard. The detection of TIPs was performed using an improved version of SPLITREADER^58^. Our SPLITREADER (vbeta2.5) pipeline uses the information of both split- and discordant reads to call non-reference insertions. In addition, we genotyped the absence of non-reference TE insertions by analyzing local coverage around the insertion sites. SPLITREADER has four steps: (i) extraction of reads mapping discordantly or not at all to the reference tomato genome, (ii) mapping to a collection of reference TE sequences and selection of the reads aligning partially or discordantly, (iii) remapping selected reads to the reference genome sequence, and (iv) identification of a cluster of split- and/or discordant reads indicating the presence of a non-reference TE insertion. Specifically, for each tomato accession, we retrieved reads that did not map to the reference genome sequence (containing SAM flag 4) or that mapped discordantly (paired reads mapping to different chromosomes or to positions separated by more than ten times the average library size). These reads were then aligned (using Bowtie2 v.2.3.2 in-local mode to allow for soft clip alignments) to a joint TE library assembled from TE annotations^23^ belonging to 467 TE families longer than 1 kbp and spanning the full range of Class I (Gypsy, Copia, and LINE) and Class II TEs (MuDR, hAT, and CACTA). Next, we selected all reads mapping to a TE sequence either partially (≥20 nt) or fully but with an unmapped mate. These reads were remapped to the tomato reference genome sequence (using Bowtie2 v.2.3.2 in-local mode to allow for soft clip alignments). Read clusters composed of at least two reads mapping in the right orientation (i.e., at least one discordant read in the +orientation upstream of the discordant read in the -orientation, or one 3′ soft-clipped read upstream of a 5′ soft-clipped read, or any combination of the cases described above) were taken to indicate the presence of a bona fide non-reference TE insertion. These sites were intersected across all accessions to identify those shared and supported in at least one individual by a minimum of three reads, including at least one upstream and one downstream. Negative coverage, as defined by the minimum WGS read depth over the upstream and downstream boundaries of a putative TE insertion site, was then calculated for each accession across all putative TE insertion sites. Accessions with negative coverage of more than five reads and lacking discordant or split-reads supporting the non-reference insertion were considered as noncarriers. Accessions with negative coverage of less than five reads and lacking discordant or split-reads supporting the non-reference insertion were considered as missing information or NA.

### Validation of TIPs

Six hundred randomly chosen TIPs detected in S_habCGN157592 (ERR418101), S_pimLYC2740 (ERR418081), S_lycPI303721 (ERR418064), S_lycEA00325 (ERR418043), and S_lycLA2706 (ERR418039) and spanning the six TE superfamilies were inspected visually using IGV, and 82% TIPs were confirmed in at least one accession. Moreover, visual inspection across 516 accessions of 56 TE insertions confirmed visually in at least one carrier genome (i.e., 28,896 visual inspections) identified 287 cases of insertions being missed (i.e., false negatives) and none being wrongly called (i.e., false positives). Also, the presence/absence of two TIPs were assessed by PCR using gDNA extracted from 22 tomato accessions, and their status was confirmed in each case (Supplementary Fig. 6). gDNA was extracted using the CTAB method, and PCRs performed with Taq DNA polymerase (NEB) using the following primers: PPEAT-For1 (GGACACCGCGGAGTAAGAAA) + PPEAT-Rev1 (GACTAGACCACGTCAAGCCC), PPEAT-For2 (TTGGAGGCGCCTGATTTCTT) + PPEAT-INS-Rev1 (TCAAGGCATTCAACAGTTGTTTTG), PSY1-For1 (ACTCCATCTGGAGAACGGAC) + PSY1-Rev1 (CATGGAATCAGTCCGGGAGG), and PSY1-For2 (CATGGAATCAGTCCGGGAGG) + PSY1-INS-Rev3 (GACCCCCGTCCTTTCTGTTT). To further assess the specificity of our SPLITREADER pipeline, we evaluated the presence of TIPs detected in the M82 cultivar on the high-quality assembled genome sequence recently obtained using Nanopore long-reads available for this accession^59^. Specifically, 1-kb sequence upstream and downstream of TIPs detected in M82 by our SPLITREADER pipeline were extracted from the Heinz 1706 reference genome (version SL2.5) and aligned using BLAT to the high-quality genome of M82. Consistent with the validation based on visual inspection, more than 70% of TIPs detected in M82 were also found in the reference M82 genome, with *COPIA* insertions showing the highest specificity (77%) (Supplementary Fig. 7). Furthermore, this estimated rate is similar to the one we obtained using the same pipeline to analyze numerous *A. thaliana* resequenced genome data and which we could validate experimentally using TE sequence capture^27,58^. Using this last dataset, we estimated that the false-negative rate of our SPLITREADER approach is about 20% overall, the highest sensitivity being achieved for TIPs belonging to the *COPIA*, *MuDR*, and *CACTA* families^58^. These rates of FP and FN are similar to those reported by others using multiple software developed to detect TIPs based on Illumina short reads^60^.

### SNP calling and phylogenetic analyses

Illumina resequencing data were aligned to the tomato genome reference v.2.50 using Bowtie2 v.2.3.2 with default parameters. The resulting alignment files were filtered to remove reads mapping to multiple locations using samtools with parameter -q 5, and to remove duplicated reads with Picard MarkDuplicates with default parameters (parameter REMOVE_ DUPLICATES = true). Finally, indels were realigned using GATK v4.1.8.0 RealignerTargetCreator and IndelRealigner successively with default parameters. Alignment files were used to call SNPs. For this, we ran GATK’s UnifiedGenotyper with default parameters in all 602 accessions simultaneously. We extracted SNPs at 8,760 positions genotyped in the SolCAP Infinium Chip SNP microarray as indicated in the tomato annotation (ITAG2.4_solCAP.gff3). We obtained a final matrix of 1,812 SNPs after removing ambiguous SNPs and SNPs in high-linkage disequilibrium using PLINK v1.90b6.9 with parameters—mind 0.1—geno 0.1—indep 50 5 3.5. A phylogenetic tree was estimated from the final matrix using the ape package in R v.3.4.4 and the neighbor-joining method including *S. pennellii* LA0716 as an outgroup. The resulting tree was plotted using the ggtree package v.1.4.11 in R v.3.4.4. Tomato accessions in the tree were classified manually taking into account previously described classifications and their positions in the tree relative to known classifications of species and type.

### TIP-based population differentiation

A principal component analysis (PCA) using 6906 TIPs was performed using the prcomp function from the stats package v.3.2.3 in R v.3.4.4. The first two eigenvectors were retained to create a two-dimensional plot.

### Genomic localization of TIPs and genes

A circos plot was constructed to represent the chromosomal distributions of genes and TEs, as well as the mappability of Illumina short reads. The number of genes and TEs annotated in the reference genome, as well as TIPs for the six superfamilies (*GYPSY*, *COPIA*, *LINE*, *MuDR*, *hAT*, and *CACTA*) were calculated in 500-kb windows using bedtools. To determine mappability, we aligned Heinz 1706 short-read resequencing data (SRA: SRR1572628) on the reference genome (version SL2.5). Mappability was defined as the fraction of uniquely mapped reads (MAPQ > = 10) in 10-kb windows. Gene ontology (GO) analyses were performed using AGRIGO v.1.2 [http://bioinfo.cau.edu.cn/agriGO/] and as input the Solyc ID of genes that contain a TE insertion within the limits of their annotation. The random expectation based on mappability bias was obtained by sampling a random set of 6906 uniquely mapped reads (MAPQ ≥ 10), and using as input for GO analysis the Solyc ID of genes that contain uniquely mapped reads within the limits of their annotation.

### Impact of TIPs on gene expression

Raw RNA-Seq data of tomato fruit pericarp on orange stage were obtained from ref. ^35^. Expression level per gene was calculated by mapping reads using STAR v2.5.3a63 on the tomato reference genome (version SL2.5) with the following arguments –outFilterMultimapNmax 50 –outFilterMatchNmin 30 –alignSJoverhangMin 3 –alignIntronMax 50000. Duplicated pairs were removed using picard MarkDuplicates. Counts over annotations (version ITAG2.4) were normalized using DESeq2^61^. To determine the transcriptomic impact of TIPs on nearby genes (located within 1 kb), the normalized transcript levels were compared between carriers and noncarrier accessions. Analysis was restricted to 1477 genes showing expression greater than 0 in at least one sample. Variation in full-length transcripts was calculated by comparing the ratio between the normalized number of reads that mapped downstream and upstream of a given TIP. This ratio was then compared between carriers and noncarrier accessions and binned by log2 fold changes (0.5–1.5], (1.5–2.5], [3.5–4.5], >4.5.

### Linkage-disequilibrium analyses

For each TIP, we calculated the pairwise *r*^2^ between the TIP and 300 SNPs located upstream and downstream, as well as the pairwise *r*^2^ between all the 600 SNP–SNP polymorphic sites around the TIP using PLINK v2. We then contrasted the percentage of TIP–SNPs and SNP–SNP comparisons that are in high LD (*r*^2^ > 0.4). Similar results were obtained when using *r*^2^ > 0.2 as a threshold to define polymorphisms with high LD (Supplementary Fig. 8).

### Genome-wide association studies

Phenotypic information for 17 important agronomic traits in tomato, including determinate or indeterminate growth, simple and compound inflorescences, leaf morphology, fruit color, shape, and taste for more than 150 accessions was retrieved by web data extraction. To this end, we performed a systematic web data scraping using Google search engine (googler, https://github.com/jarun/googler) followed by text pattern matching. We noted that the World Tomato Society webpage (https://worldtomatosociety.com/) compiles, in a consistent manner, phenotypic information for a large number of varieties commercialized by numerous seed banks. We thus focused our web data scraping on this webpage. Phenotypes were transformed either in boolean (1 or 0) or quantitative variables (Supplementary Data 2–17). For fruit color phenotype, accessions with red, purple–black, or pink fruits were considered as high lycopene-containing, and those with green, white, yellow, or orange fruits were considered as low lycopene-containing fruits and codified as 1 or 0, respectively. Metabolomic and volatile quantitative data from ripe fruits of 397 accessions were obtained from refs. ^25,35^. SNPs and TIPs with MAF < 1% or more than 20% of missing data were excluded. SNP-GWAS was restricted to biallelic SNPs and LD-pruned using PLINK v1.90b6.9^62^ option-indep-pairwise 50 5 0.2. GWAS was performed using linear mixed models (LMM) encoded in the software EMMAX^63^. SNP-based Kinship matrix was calculated (emmax-kin-intel64 -v -d 10) and included in the models as a random effect to control population structure and minimize false positives. Manhattan and qq plots for genome-wide association studies were performed using qqman package v.0.1.4^64^. *r*^2^ between the leading associated variant and all other associated variants in Fig. 3g was calculated using PLINK v1.90b6.9^62^ and represented by color code. Effect sizes of associations presented in Fig. 4c correspond to the beta values of the leading variant from each associated locus identified by the LMM. Given the lower sensitivity and specificity of TIP calling compared with SNPs, which could affect GWAS results, we inspected visually all associated TIPs, and we removed or corrected false-positive TIPs and negative insertion calls, respectively. Following these corrections, TIP-GWAS was performed again, and only associations with manually curated, high-confidence, TIPs were retained. In addition, the presence/absence of the two key TE insertions studied in detail (i.e., insertions within *PSY1* and *PPEAT*) was assessed by PCR using genomic DNA extracted from 22 tomato accessions, and their status was confirmed in each case (Supplementary Fig. 6). Finally, we tested the robustness of our TIP-GWAS approach by randomizing 1000 times the set of carriers and non-carrier accessions, and running GWAS for the two traits with validated associations (i.e., fruit color and potato leaf)^28,41^. In both cases, the number of permuted datasets with associations was below the family-wise significance threshold (*p* < 0.001 and *p* < 0.038, respectively; Supplementary Fig. 9).

### Local assembly of *r*^del^ allele

Visual inspection of RNA-seq coverage of accessions harboring the *r*^Del^ allele suggested a complex rearrangement. To assemble *r*^Del^ locus, WGS reads mapping concordantly or discordantly over *PSY1* locus from accessions carrying the *r*^Del^ locus were extracted and locally assembled using SPAdes V3.13.1^65^.

### Haplotype analysis

SNPs within 10 kb of the *PSY1* locus were retrieved for 602 accessions and used as input into fastPHASE^66^ version 1.4.0. Default parameters were kept, except for the -Pzp option. For each SNP, haplotype membership with the highest likelihood was assigned.

### Plant materials and growth conditions

Tomato seeds from *S. lycopersicum* cv. Heinz 1706 and TS-666 were grown in a growth chamber (Percival) using 20-l pots, 16/8-h photoperiod, 24 ± 3 °C, 60% humidity, and 200 ± 100 mmol m^−2^ s^−1^ incident irradiance. Pericarp for at least four ripe fruits from two plants was harvested 60 days after anthesis, immediately frozen in liquid N_2_, and kept at −80 °C until use.

### Full-length cDNA nanopore sequencing

Total RNA was extracted from 100 mg of ripe fruits using the Nucleo-spin RNA Plant mini kit (Macherey-Nagel). Library preparation and Nanopore sequencing were performed at the Ecole normale superieure genomic core facility (Paris, France). After checking RNA quality by Fragment Analyzer, 10 ng of total RNA was amplified and converted into cDNA using SMART-Seq v4 Ultra Low Input RNA kit (Clontech). About 17 fmol of cDNA was used for library preparation using the PCR Barcoding kit (SQK-PBK004 kit, ONT) and cleaned up with 0.6× Agencourt Ampure XP beads. About 2 fmol of the purified product was amplified during 18 cycles, with a 17-min elongation step, to introduce barcodes. Samples were multiplexed in equimolar quantities to obtain 20 fmol of cDNA, and the rapid adapter ligation step was performed. Multiplexed library was loaded on an R9.4.1 flowcell (ONT) according to the manufacturer’s instructions. A standard 72-h sequencing was performed on a MinION MkIB instrument. MinKNOW software (version 19.12.5) was used for sequence calling. Long-reads were mapped on the tomato reference genome (SL2.5) using minimap2^67^ V2.11-r797 and visualized with IGV.

### Reporting summary

Further information on research design is available in the Nature Research Reporting Summary linked to this article.

## Supplementary information

Supplemnetary Information

Peer Review File

Reporting Summary

Description of Additional Supplementary Files

Supplementary Data 1

Supplementary Data 2

Supplementary Data 3

Supplementary Data 4

Supplementary Data 5

Supplementary Data 6

Supplementary Data 7

Supplementary Data 8

Supplementary Data 9

Supplementary Data 10

Supplementary Data 11

Supplementary Data 12

Supplementary Data 13

Supplementary Data 14

Supplementary Data 15

Supplementary Data 16

Supplementary Data 17

## Data Availability

Data supporting the findings of this work are available within the paper and its Supplementary Information files. A reporting summary for this article is available as a Supplementary Information file. All datasets generated and analyzed during the current study are available from the corresponding authors upon request. Long-read nanopore sequencing data have been deposited in the European Nucleotide Archive (ENA) under project PRJEB37834. Short-read sequencing data of tomato genomes reanalyzed in this study have been obtained from ENA under projects PRJNA259308, PRJEB5235, and PRJNA353161. The tomato reference genome (*Solanum lycopersicum* cv. Heinz, release SL2.5) used in this study was obtained from SOL genomics [ftp://ftp.solgenomics.net/tomato_genome]. The source data underlying Figs. 1a, f, 2c, d, 3d, e, j, 4a, b, d, 5d, e, h, i as well as Supplementary Fig. 3a are provided as a Source Data file. Source data are provided with this paper.

## Source data

Source Data

## Code availability

Codes used to detect TE insertions are available at GitHub [https://github.com/baduelp/public]. Source data are provided with this paper.
