# Author Correction: The impact of transposable elements on tomato diversity

**DOI:** 10.1038/s41467-021-23578-y

**Published:** 2021-05-21

**Authors:** Marisol Domínguez, Elise Dugas, Médine Benchouaia, Basile Leduque, José M. Jiménez-Gómez, Vincent Colot, Leandro Quadrana

**Affiliations:** 1 Institut de Biologie de l’Ecole Normale Supérieure (IBENS), Centre National de la Recherche Scientifique (CNRS), Institut National de la Santé et de la Recherche Médicale (INSERM), Ecole Normale Supérieure, PSL Research University, 75005 Paris, France; 2 Genomic facility, Institut de Biologie de l’Ecole Normale Supérieure (IBENS), Département de biologie, École normale supérieure, CNRS, INSERM, Université PSL, 75005 Paris, France; 3 Institut Jean-Pierre Bourgin, INRAE, AgroParisTech, Université Paris-Saclay, 78000 Versailles, France

**Keywords:** Agricultural genetics, Genome-wide association studies, DNA transposable elements, Natural variation in plants

Correction to: *Nature Communications* 10.1038/s41467-020-17874-2, published online 13 August 2020.

The original version of this Article included an incorrect Source Data file, in which the Source Data of Fig. 5D was incorrectly sorted. The HTML has been updated to include a corrected version of Source Data file. The original incorrect version of Source Data file can be found as Supplementary Information associated with this Correction.

## Supplementary information

### Source data

Source data file

