# Supplementary material for: The impact of transposable elements on tomato diversity
Source: Nat Commun. 2020 Aug 13;11:4058. doi: 10.1038/s41467-020-17874-2 (PMC7426864; doi:10.1038/s41467-020-17874-2)
Supplement: Supplementary file 1 — Supplemnetary Information [file 41467_2020_17874_MOESM1_ESM.pdf]

The impact of transposable elements on tomato diversity

Dominguez *et al.*

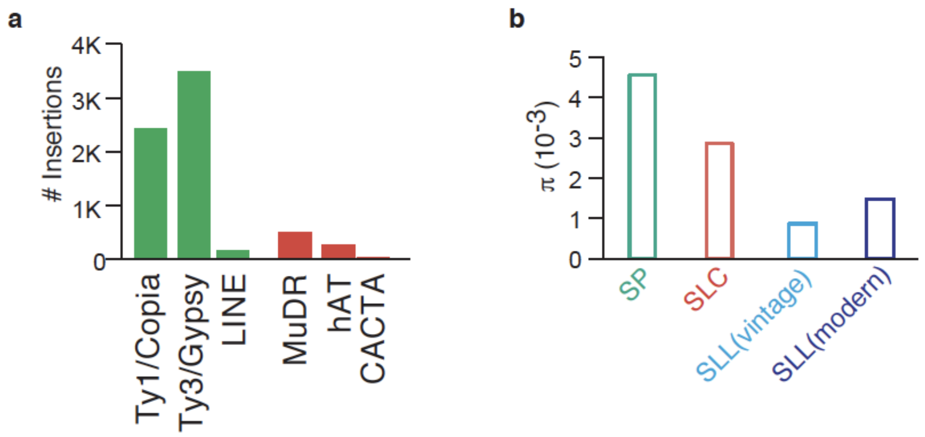

Supplementary Figure 1. The tomato mobilome and genetic diversity per group. a. Number of TIPs per TE superfamily, in green Class I LTR and non-LTR retroelements, and in red DNA transposons. **b**.Genetic diversity per tomato group.

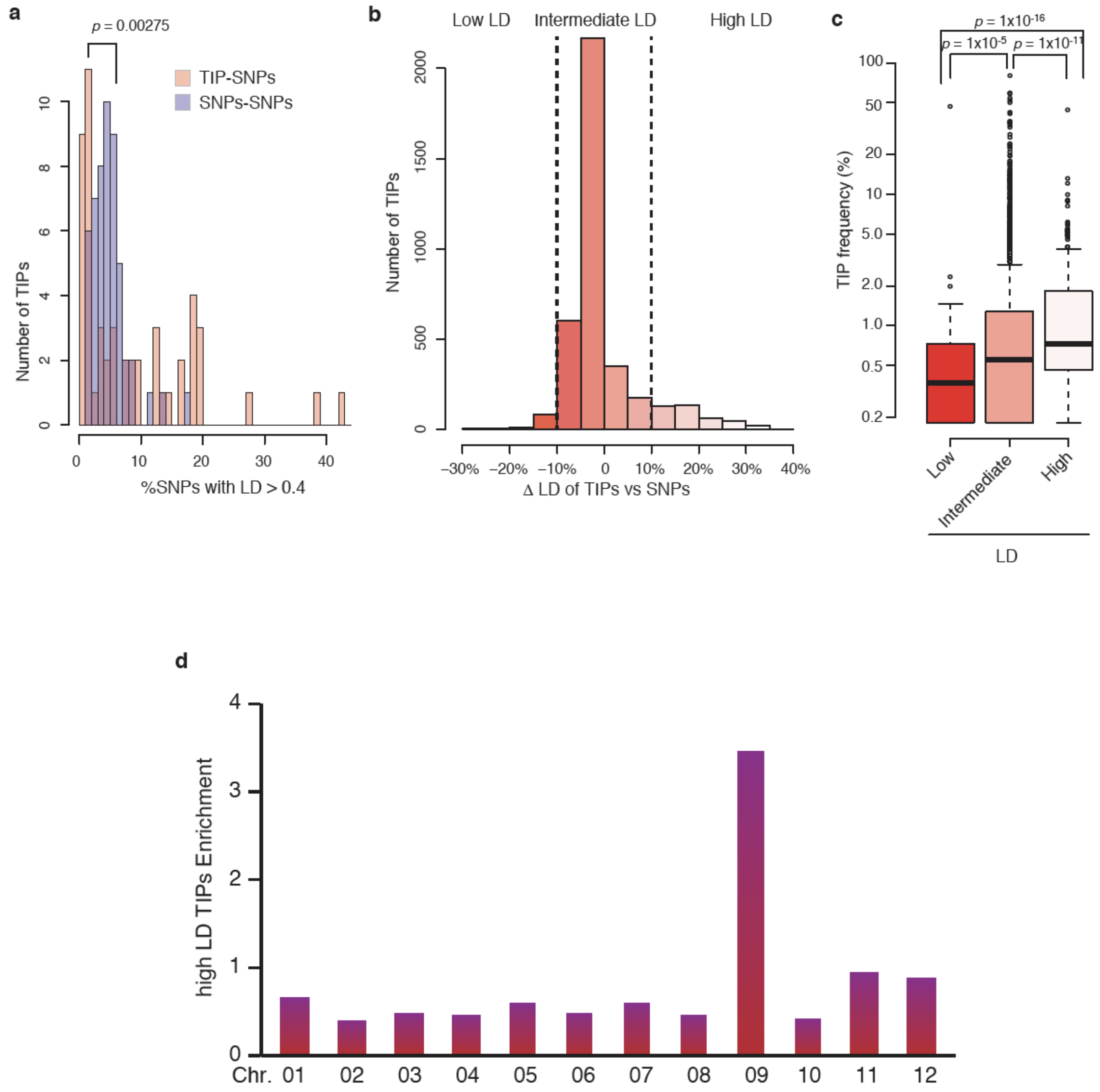

Supplementary Figure 2. LD between TIPs and SNPs. a. Distribution of the percentage of SNPs tagging (LD>0.4) TIPs (red) and SNPs (blue). Only visually validated TIPs were considered in this analysis (n= 56). Statistical significance for differences was obtained using one-sided paired t-test. **b**. Distribution of the proportion of SNPs that are in lower or higher linkage disequilibrium (LD) with TIPs or other SNPs. **c**. LD between TIPs and SNPs in relation to TIPs frequency. For each boxplot, the lower and upper bounds of the box indicate the first (Q1) and third (Q3) quartiles, respectively, and the center line indicates the median and the whiskers represent data range, bounded to 1.5 * (Q3-Q1). Statistical significance for differences was obtained using two-sided MWU test. **c**. Enrichment of high LD TIPs per chromosome.

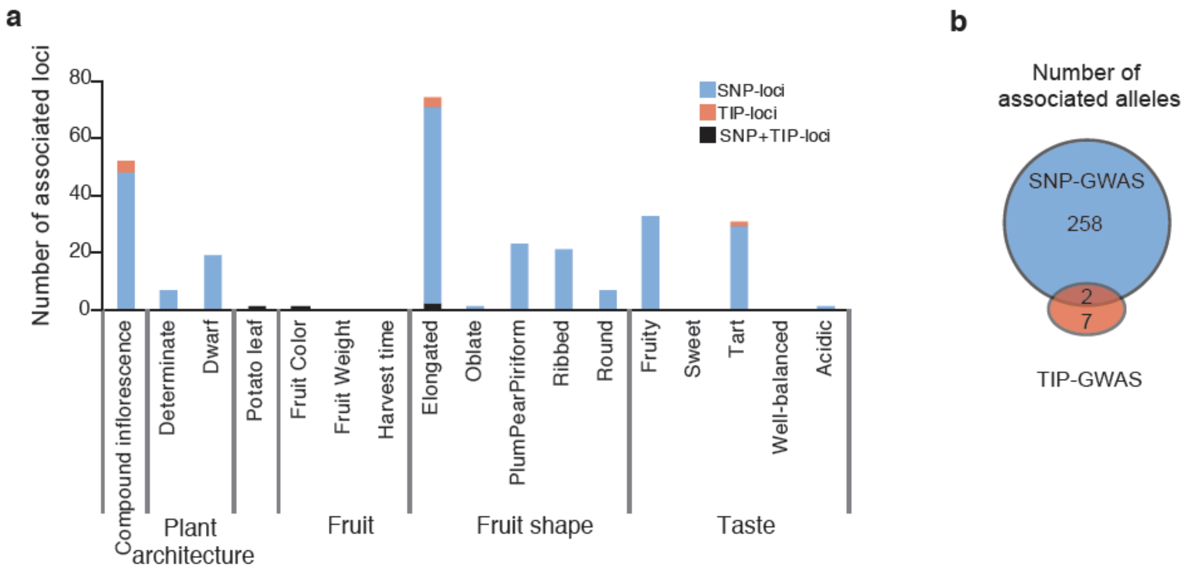

Supplementary Figure 3. TIP- and SNP-GWAS of agronomically important traits. a. Number of loci associated with variation in 17 traits. loci detected by both SNPs and TIPs are indicated in black. **b.** Significant associations detected by SNP- and TIP-GWAS and their overlap. Source data of Supplementary Fig. 3a is provided as a Source Data file.

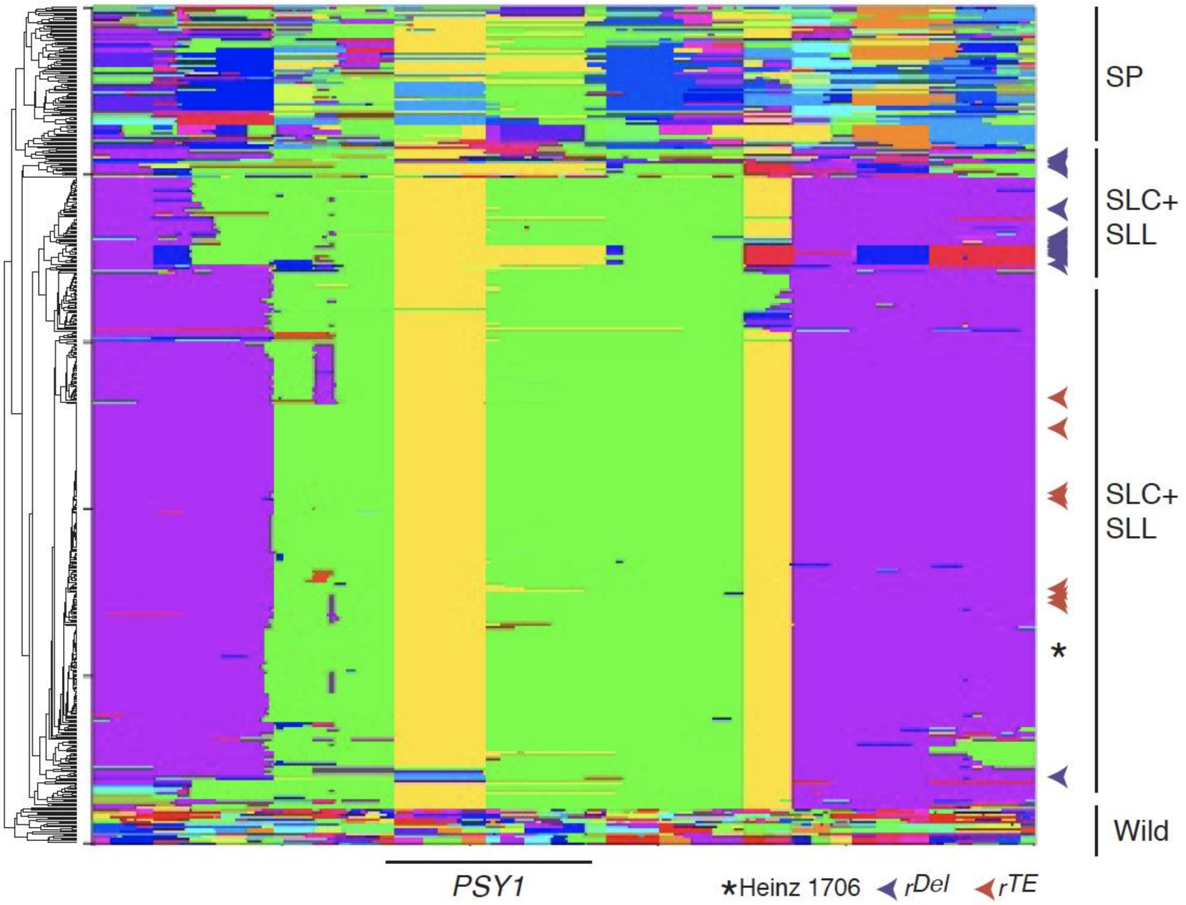

Supplementary Figure 4. Reconstruction of *PSY1* haplotypes. Distinct haplotype blocks around *PSY1* are represented by different colors.

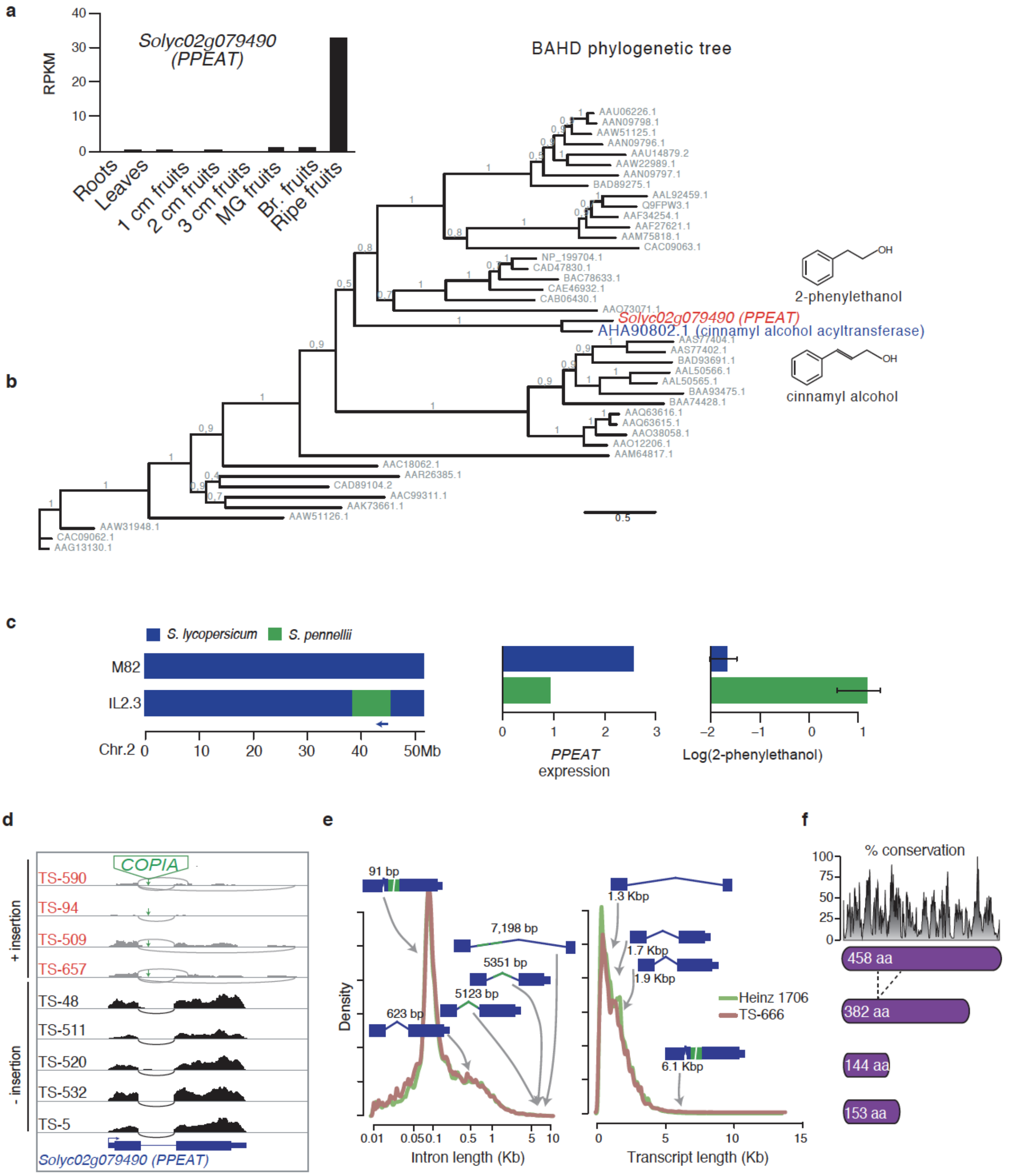

Supplementary Figure 5. *Solyc02g079490* encodes a putative 2-phenylethanol Acyl-CoA transferase. **a**. Expression profile of *Solyc02g079490* in leaves and during fruit development. **b.** phylogenetic tree of the characterized plant BAHD family of acyltransferases. **c**. Genotype, *Solyc02g079490* expression and 2-phenylethanol levels in M82 plants and an IL harboring the wild allele of *PPEAT*. Error bars represent SD of 2-phenylethanol quantification by gas chromatography of a single pool of two fruits. **d**. Genome browser view of RNA-seq coverage over *Solyc02g079490* of accessions carrying or not the associated TE insertion. **e**. Intron and transcript length distribution based on our Nanopore transcriptomic data. *PPEAT* transcripts are indicated. **f**. % conservation of PPEAT based on the alignment of 45 plant BAHD acyltransferases (from b).

PPEAT

TE insertion absence (PPEAT-For1 + PPEAT-Rev1)

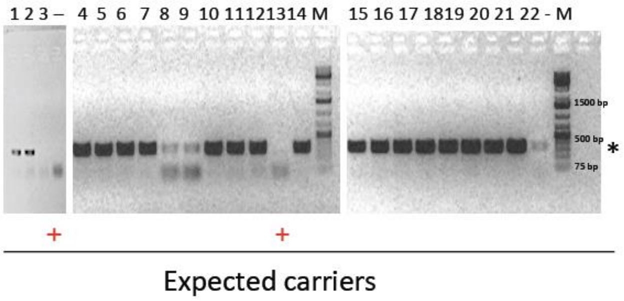

TE insertion presence (PPEAT-For2 + PPEAT-INS-Rev1)

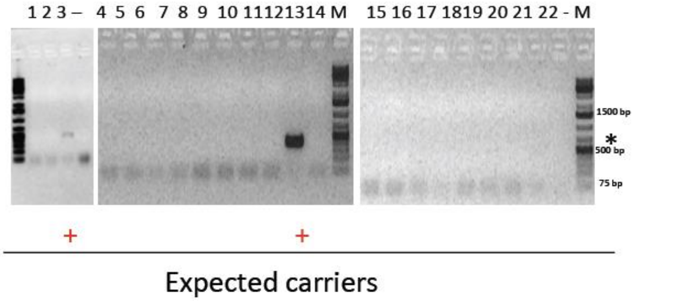

PSY1

TE insertion absence (PSY1-For1 + PSY1-Rev1)

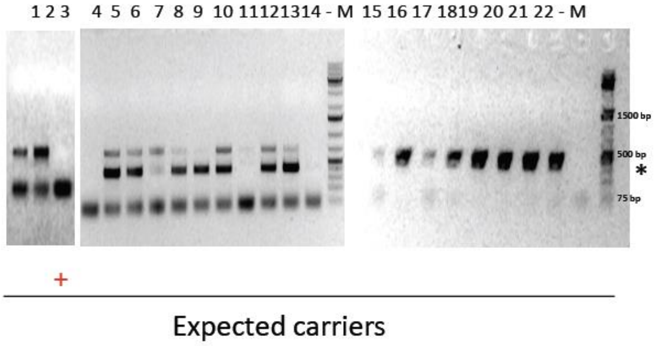

TE insertion presence (PSY1-For2 + PSY1-INS-Rev3)

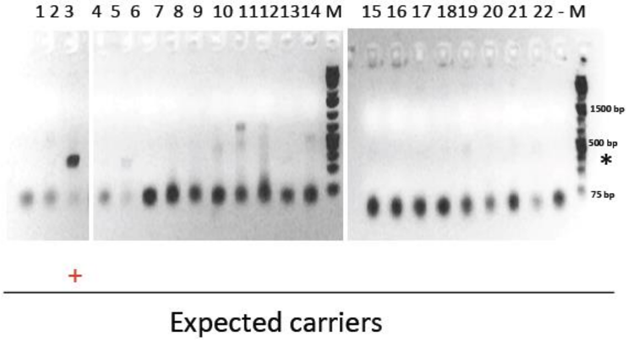

line	sample
1	Heinz 1706
2	TS-666
3	TS-528
-	water
4	LA0400/TS265
5	LA0722/TS123
6	LA1245/TS124
7	LA1246/TS016
8	LA1617/TS-145
9	BGV006775
10	LA1478/TS266
11	LA1547/TS014
12	LA1578/TS437
13	LA1582/TS092
14	LA0417/TS295
M	1kb+ marker
15	LA1237/TS077
16	BGV007931
17	LA1307/TS057
18	LA1388/TS66
19	EA00990/TS681
20	EA01037/TS735
21	EA01155/TS177
22	LYC1410/TS696/HeinzES 58
-	water
M	1kb+ marker

Supplementary Figure 6. PCR-based validation of TIPs within *PPEAT* and *PSY1*. Genotyping of TE insertion presence/absence by PCR. * indicated the position of the expected band. PCR-based genotypes were confirmed at least twice.

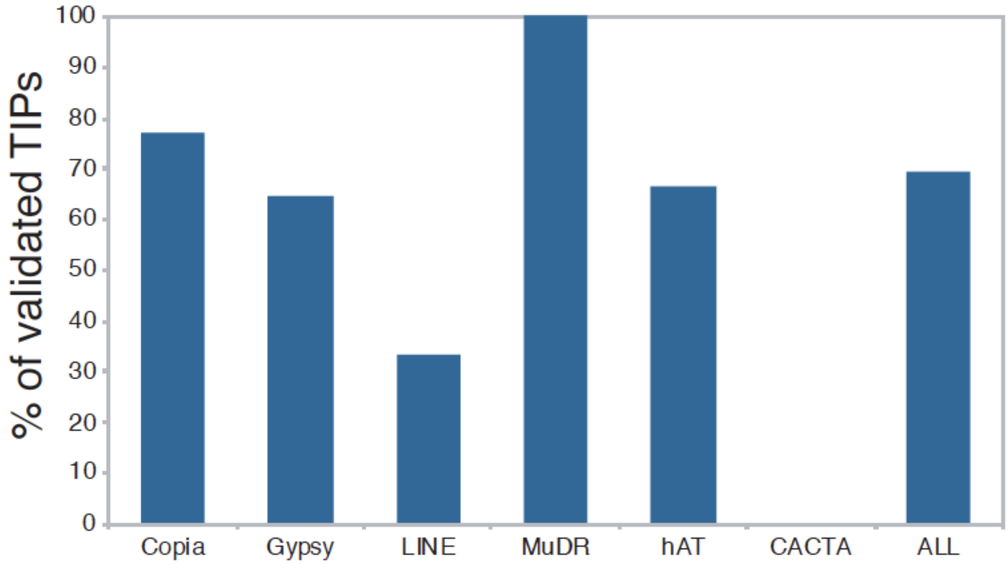

Supplementary Figure 7. Specificity of the SPLITREADER pipeline.

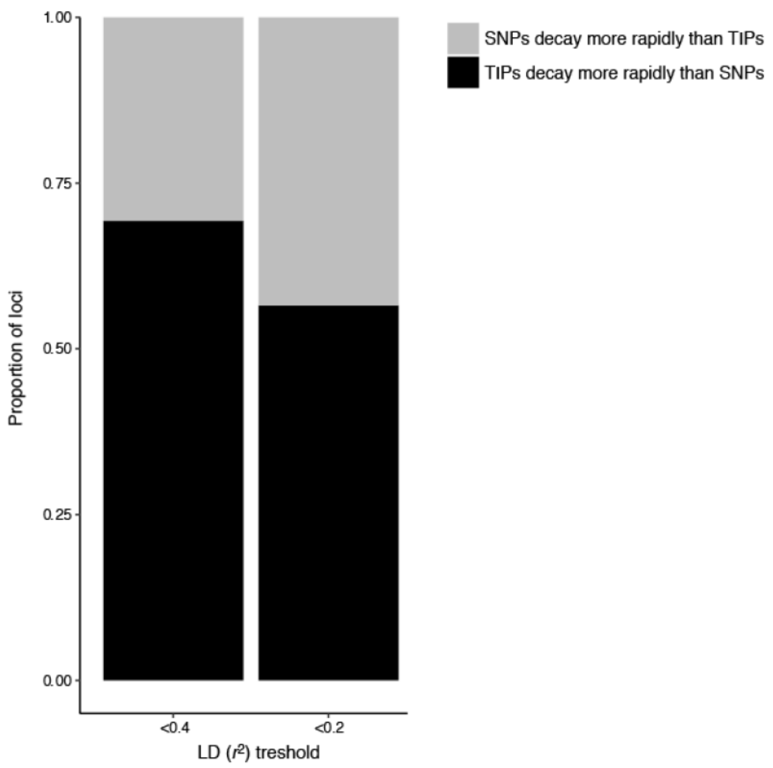

Supplementary Figure 8. LD between TIPs and SNPs using different r^2 cutoffs. The proportion of loci (i.e. TIPs) having less SNPs in high LD (r^2>0.4 and r^2>0.2) than their corresponding SNPs.

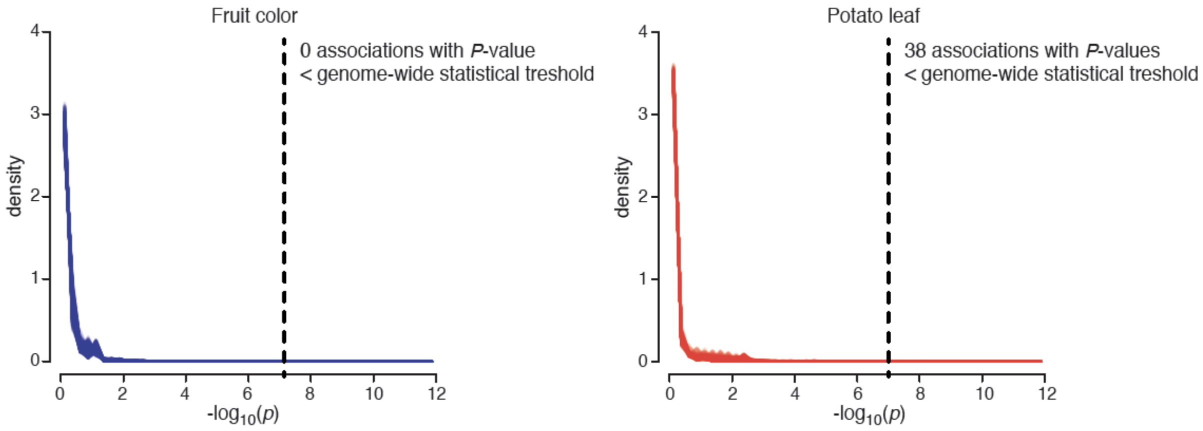

Supplementary Figure 9. Empirical distribution of TIP-GWAS *p* values obtained under the null-hypothesis. Genotypes:traits pairs were randomized 1000 times and analyzed by LMM model. Each blue and red line represents the result of LMMs obtained with randomized genotypes:traits pairs. Dashed line indicates the threshold (corresponding to a bonferroni-corrected alfa value = 0.05) used to detect statistical differences.
