# Supplementary material for: The impact of transposable elements on tomato diversity
Source: Nat Commun. 2020 Aug 13;11:4058. doi: 10.1038/s41467-020-17874-2 (PMC7426864; doi:10.1038/s41467-020-17874-2)
Supplement: Supplementary file 3 — Reporting Summary [file 41467_2020_17874_MOESM3_ESM.pdf]

nature research

Corresponding author(s): Vincent Colot
Leandro Quadrana

Last updated by author(s): Jul 19, 2020

Reporting Summary

Nature Research wishes to improve the reproducibility of the work that we publish. This form provides structure for consistency and transparency in reporting. For further information on Nature Research policies, see Authors & Referees and the Editorial Policy Checklist .

Statistics

For all statistical analyses, confirm that the following items are present in the figure legend, table legend, main text, or Methods section.

n/a	Confirmed	
☐	☒	The exact sample size (*n*) for each experimental group/condition, given as a discrete number and unit of measurement
☐	☒	A statement on whether measurements were taken from distinct samples or whether the same sample was measured repeatedly
☐	☒	The statistical test(s) used AND whether they are one- or two-sided<br>*Only common tests should be described solely by name; describe more complex techniques in the Methods section.*
☐	☒	A description of all covariates tested
☐	☒	A description of any assumptions or corrections, such as tests of normality and adjustment for multiple comparisons
☐	☒	A full description of the statistical parameters including central tendency (e.g. means) or other basic estimates (e.g. regression coefficient) AND variation (e.g. standard deviation) or associated estimates of uncertainty (e.g. confidence intervals)
☐	☒	For null hypothesis testing, the test statistic (e.g. *F*, *t*, *r*) with confidence intervals, effect sizes, degrees of freedom and *P* value noted<br>*Give P values as exact values whenever suitable.*
☒	☐	For Bayesian analysis, information on the choice of priors and Markov chain Monte Carlo settings
☒	☐	For hierarchical and complex designs, identification of the appropriate level for tests and full reporting of outcomes
☐	☒	Estimates of effect sizes (e.g. Cohen's *d*, Pearson's *r*), indicating how they were calculated

Our web collection on statistics for biologists contains articles on many of the points above.

Software and code

Policy information about availability of computer code

Data collection	Data collection for Nanopore sequencing was performed with MinKNOW software (versions 19.12.5).<br>phenotypic information for 17 important agronomic traits in tomato, including determinate or indeterminate growth, simple and compound inflorescences, leaf morphology, fruit color, shape and taste for more than 150 accessions was retrieved by web data scraping using google search engine (googler; https://github.com/jarun/googler) followed by text pattern matching.
Data analysis	SPLITREADER vbeta2.5 (Quadrana et al., 2016): available at https://github.com/baduelp/public<br>Bowtie2 v2.3.2 (Langmead and Salzberg, 2012): available at https://sourceforge.net/projects/bowtie-bio/files/bowtie2/2.3.5.1/<br>GATK v4.1.8.0 (Poplin et al. 2017): available at https://github.com/broadinstitute/gatk/releases<br>STAR v2.5.3a (Dobin et al., 2013): available at https://github.com/alexdobin/STAR<br>DESeq2 (Love et al., 2014): available at https://bioconductor.org/packages/release/bioc/html/DESeq2.html<br>Picard Tools (available at https://broadinstitute.github.io/picard/)<br>samtools v1.2.1 (Li et al., 2009): Available at http://www.htslib.org/download/<br>EMMAX vintel64-20120205 (Kang et al 2010): Available at http://csg.sph.umich.edu//kang/emmax/download/index.html<br>PLINK v2 (Purcell et al 2007): Available at http://s3.amazonaws.com/plink2-assets/plink2_linux_x86_64_20200310.zip<br>PLINK v1.90b6.9 (Purcell et al 2007): Available at http://s3.amazonaws.com/plink2-assets/plink2_linux_x86_64_20200310.zip<br>SPAdes V3.13.1 (Nurk et al 2013): Available at http://cab.spbu.ru/files/release3.13.1/SPAdes-3.13.1-Linux.tar.gz<br>fastPHASE48 V1.4.0 (Scheet et al., 2006): Available at http://scheet.org/software.html<br>MinKNOW v19.12.5: Available at https://nanoporetech.com/nanopore-sequencing-data-analysis<br>minimap2 v2.11-r797 (Li, H. et al 2018): Available at https://github.com/lh3/minimap2

For manuscripts utilizing custom algorithms or software that are central to the research but not yet described in published literature, software must be made available to editors/reviewers. We strongly encourage code deposition in a community repository (e.g. GitHub). See the Nature Research guidelines for submitting code & software for further information.

Data

Policy information about availability of data

All manuscripts must include a data availability statement. This statement should provide the following information, where applicable:

- Accession codes, unique identifiers, or web links for publicly available datasets
- A list of figures that have associated raw data
- A description of any restrictions on data availability

Long-read nanopore sequencing data has been deposited in the European Nucleotide Archive (ENA) under project PRJEB37834 [https://www.ebi.ac.uk/ena/data/view/PRJEB37834]. Short-read sequencing data of tomato genomes reanalyzed in this study have been obtained from ENA under projects PRJNA259308 [https://www.ebi.ac.uk/ena/data/view/PRJNA259308], PRJEB5235 [https://www.ebi.ac.uk/ena/data/view/PRJEB5235] and PRJNA353161 [https://www.ebi.ac.uk/ena/data/view/PRJNA353161]. Tomato reference genome (Solanum lycopersocum cv. Heinz, release SL2.5) used in this study was obtained from SOL genomics [ftp://ftp.solgenomics.net/tomato_genome]. A reporting summary for this Article is available as a Supplementary Information file. All datasets generated and analyzed during the current study are available from the corresponding author upon request. The source data underlying Fig. 1a, 1f, 2c, 2d, 3d, 3e, 3j, 4a, 4b, 4d, 5d, 5e, 5h, 5i and Supplementary Figure. 3a are provided as a Source Data file.

Field-specific reporting

Please select the one below that is the best fit for your research. If you are not sure, read the appropriate sections before making your selection.

- [x] Life sciences
- [ ] Behavioural & social sciences
- [ ] Ecological, evolutionary & environmental sciences

For a reference copy of the document with all sections, see nature.com/documents/nr-reporting-summary-flat.pdf

Life sciences study design

All studies must disclose on these points even when the disclosure is negative.

Sample size	Previously published data sets were used as they are. Nanopore sequencing of cDNA was performed on the reference genome accession and an additional accession picked randomly from the set of accessions that carry the intronic insertion in PPEAT
Data exclusions	No data were excluded
Replication	Nanopore sequencing was performed on RNA extracted from a pool of at least four ripe fruits collected from two plants. PCR-based genotyping was performed from DNA extracted from pool of leaves from at least three plants and PCRs performed at least twice.
Randomization	Previously published data sets were used as they are. Nanopore sequencing of cDNA was performed on the reference genome accession and one additional accession was selected randomly from the set of accessions carrying the intronic insertion in PPEAT. Accessions used for PCR-based genotyping were selected randomly.
Blinding	Previously published data sets were used as they are. The investigators were blinded to group allocation during TIPs detection, web-based phenotypic data collection as well as GWAS and transcriptomic analyses. The investigators were not blinded to accession's name during sample collection for genotypic and long-read transcriptomic analyses.

Reporting for specific materials, systems and methods

We require information from authors about some types of materials, experimental systems and methods used in many studies. Here, indicate whether each material, system or method listed is relevant to your study. If you are not sure if a list item applies to your research, read the appropriate section before selecting a response.

Materials & experimental systems

n/a	Involved in the study	
x		Antibodies
x		Eukaryotic cell lines
x		Palaeontology
x		Animals and other organisms
x		Human research participants
x		Clinical data

Methods

n/a	Involved in the study	
x		ChIP-seq
x		Flow cytometry
x		MRI-based neuroimaging
