# Supplementary material for: The impact of transposable elements on tomato diversity
Source: Nat Commun. 2020 Aug 13;11:4058. doi: 10.1038/s41467-020-17874-2 (PMC7426864; doi:10.1038/s41467-020-17874-2)
Supplement: Supplementary file 4 — Description of Additional Supplementary Files [file 41467_2020_17874_MOESM4_ESM.pdf]

Description of Additional Supplementary Files
:
File name: Supplementary Data 1
Description: TIPs presence/absence across 602 tomato accessions

File name: Supplementary Data 2
Description: Potato leaf morphology

File name: Supplementary Data 3
Description: Determinate or indeterminate growth morphology

File name: Supplementary Data 4
Description: Simple or compound inflorescence morphology

File name: Supplementary Data 5
Description: Dwarf/normal plant architecture

File name: Supplementary Data 6
Description: Harvest time

File name: Supplementary Data 7
Description: Elongated fruit shape

File name: Supplementary Data 8
Description: Oblate fruit shape

File name: Supplementary Data 9
Description: Plum/pear/periform fruit shape

File name: Supplementary Data 10
Description: Ribbed fruit shape

File name: Supplementary Data 11
Description: Round fruit shape

File name: Supplementary Data 12
Description: Mature fruit weight

File name: Supplementary Data 13
Description: Acidic fruit taste

File name: Supplementary Data 14
Description: Fruity fruit taste

File name: Supplementary Data 15
Description: Sweet fruit taste

File name: Supplementary Data 16
Description: Tart fruit taste

File name: Supplementary Data 17
Description: Well-balanced fruit taste
