# Supplementary material for: The impact of transposable elements on tomato diversity
Source: Nat Commun. 2020 Aug 13;11:4058. doi: 10.1038/s41467-020-17874-2 (PMC7426864; doi:10.1038/s41467-020-17874-2)
Supplement: Supplementary file 2 — Peer Review File [file 41467_2020_17874_MOESM2_ESM.pdf]

Reviewers' comments:

Reviewer #1 (Remarks to the Author):

In the manuscript under consideration, the authors have presented their discovery of TEs/TIPs in tomato genome and their contributions to expression and phenotypic variance. I have significant concerns towards the validity of the GWAS and LD analysis.

The main concern is the quality of the input data of TE/TIPs. Unlike other analyses (such as evolution or impact to gene expression), GWAS replies on an accurate estimate of allele frequencies in the focal population. The repetitiveness in the TE related regions brings obstacles to such efforts. If we set the parameters to call TE/TIPs aggressively, it will bring false positives; however a rigorous filtering will lead to high false negatives, which hurt the analyses equality as false positives do.

In the current version of the manuscript, the FP and FN have not been thoroughly analyzed. Regarding FN, it is stated: "To limit false-negatives, non-carrier individuals with less than five reads negative coverage were considered as missing information or NA". This statement doesn't quantitatively show how large is the FN in the resulting dataset. For FP, it is stated: "we evaluated the presence of TIPs detected in the M82 cultivar on the high-quality assembled genome available for this accession. Consistent with the validation based on visual inspection, nearly 70% of TIPs detected in M82 were also found in the reference M82 genome, with COPIA insertions showing the highest specificity (77%)". I have two concerns of this statement. The percentage of 70% or 77% looks quite low for me: a 30% or 23% of FP will make the estimate of allele frequencies quite distorted for both GWAS and LD calculations, not to mention the unknown FN. Second, after reading the reference 42, that has generated the "high-quality assembled genome", I find that the TEs are not their focus, although it makes sense for me to use third-generation sequencing to assess TEs. We need more rigorous analyses of how is the quality of TE/TIPs reported by that paper.

Because of the above limitation, the LD calculation between TEs and SNPs are also not convincing. Indeed, they found the LD between SNPs is higher than LD between TE and SNPs. This is actually consistent to my observations in other plant genomes, indicating high false negatives in the data related of structural variants. Assuming most SNPs and TEs are not functionally neural, I'd expect in general these two are similar (although some TEs or SNPs are in high LD with others because of selections). However the current short reads can't offer good quality of calls in terms of both FP and FN. As such, the LD calculations may not support their claim regarding SNPs can't tag the contributions of TEs towards the phenotypic variance.

To justify the validity of the work, the authors may conduct rigorous experimental validations to quantify the FP/FN of their TE/TIPs discoveries. Alternatively, a functional validation of the GWAS peaks may also serve as an evidence of the ultimate findings.

Minor issues:

Page 18: The word ambiguous appears twice at line 393.

Page 19: There is no citation regarding where they get the fruit color phenotype for 348 accessions. It might be helpful to provide more details on which seed banks databases they are using, what the raw data look like, and how they process the data.

Page 20: Why only 10 permutations? This sounds a bit low for getting a random expectation.

As the minimal requirement in presentations of a GWAS signal, one may provide a full Manhattan plot (instead of only 1.2Mb in Chromosome 3 in your figure 3 or 2Mb in Chromosome 2 in your figure 5) to show the overall picture of the genetic architecture of the trait. Also, a QQ plot of expected and

observed P-values needs to be included to justify that there is no inflation of the distribution of P-values.

Reviewer #2 (Remarks to the Author):

Review of “The impact of transposable elements on tomato diversity”

This manuscript examines transposable element insertion polymorphisms in previously sequenced tomato lines, examining several aspects of these polymorphisms. In brief, while some analyses presented here are interesting, the scholarship left a lot to be desired and too many results were un- or under-explored.

Regarding the scholarship, there are many papers that have examined TE polymorphisms previously, including in plants and including looking at associations with gene expression. These are far from being “ignored from most population genomic studies” (line 176). To name a few papers that examine TEs, these include in rice (Carpentier et al. 2019, Nature Communications), Arabidopsis (Stuart et al. 2016, eLife), Brachypodium (Eichten et al. 2016, Genome Research), carrot (Macko-Podgórni et al. 2019, Mobile DNA), Arabis (Rogivue et al. 2019, Molecular Ecology Resources), and multiple species of Capsella (Agren et al. 2016, Am J Bot; Uzunovic et al. 2019, Molecular Biology and Evolution). Though none of these are cited in the current submission, these are largely not obscure papers. In fact I can see that the current authors have cited some of these papers elsewhere, and for good reason: many of the same patterns they find in tomatoes are found in these species. For instance, in reviewing several of these studies, Gaut et al. (2018, Nature Plants) noted that “two-thirds of transposable elements (TEs) in a population survey of Arabidopsis thaliana were not in linkage disequilibrium with a nearby SNP”.

In addition, there are just as many similar studies in animal systems (especially Drosophila and humans) that deserve mention and that should be read for their careful consideration of many of the issues encountered here (e.g. Cridland et al. 2015, Genetics). Similarly, the idea that structural variants (including TEs) may help to improve GWAS and are often not tagged by SNPs has been a major focus of human studies for over a decade (Manolio et al. 2009, Nature).

Overall, this manuscript would have been a lot better had it been placed in context within the field, exploring the patterns presented rather than applauding them.

Some specific comments about results presented in the manuscript:

-The authors report a different distribution of non-reference TE insertion polymorphisms relative to reference TEs. But I have to think that the way in which TEs were mapped to specific positions in the genome would ensure that this would always be the case. Because the tomato genome has large regions covered by TEs, the requirement for unique mapping of TE polymorphisms both upstream and downstream (lines 368-371) would automatically exclude them from being mapped to highly repetitive regions. Exploring the “mappability” of TEs to specific parts of the genome would help to put these distributions in context. Additionally asking whether specific types of genes were in regions that were more mappable than others would also help to make specific GO-term overrepresentation more convincing as a biological pattern.

-Lines 88-90: “TIPs associated with two-fold or more changes in gene expression are proportionally more frequent when located in exons and introns (43% and 37%, respectively) than in other gene

compartments (Fig. 2d).” I actually don’t see this in the figure. The light blue and dark blue bars seem to be the same height in all compartments.

-Lines 114-115: Are there fewer TEs in high LD with SNPs because of their lower average population frequency? If you frequency-match TEs and SNPs, is there still lower LD? Why would this be the case?

Reviewer #3 (Remarks to the Author):

The manuscript submitted by Dominguez et al. reports the identification of transposable elements insertion polymorphisms in tomato and a GWAS analysis to tentatively identify association between some TIPs and agronomic traits in this crop (fruit quality traits). The paper is well written, the study is of interest to plant genomicists and the functional impact of structural variations in plants is definitely a trendy topic. I have, however, several major concerns about this manuscript the way it stands :

1 – The functional relationships between TIPs and fruit quality (whether identified using a candidate gene approach or through GWAS) are tentative. The authors do not provide any functional validation of what they claim and no causal relationship is secured for any of the traits (except for the association of a TIP with fruit colour, used here as positive control). Therefore, most of the results presented by the authors are over-interpreted and the result section should be rewritten. In particular, the claims made in paragraph p6, l01-110 are pure speculation. This should be removed or rephrased.

2 – The authors have used a « refined version of SPLITREADER » . What does this exactly mean ? Why did they have to refine their existing software ? Was it because it was originally designed for a 100Mbp genome and did not work for the much larger genome of tomato ? If yes it should be mentioned. What was changed (« refined ») ? In addition, the authors have completely ignored the existing literature on the subject of TIPs detection from NGS data. This should be corrected. For instance Vendrell-Mir et al. (Mob. DNA 2019) have conducted a major comparative analysis of existing softwares. How does the « refined » version of SPLIREADER compare to these ? In addition, a similar survey was conducted on rice (Carpentier et al., 2019). This work is not cited, which considerably weakens the present manuscript.

3 – This work is not the first report of the use of TIPs-GWAS. It was reported on Brassica napus (Song et al., Nature Plant, 2020) and rice (Fuentes et al., Genome Research 2019 ; Akakpo et al. , New phytologist 2020). Again, the authors should have acknowledged it and compared and discussed their results with the previously published ones on two other crops.

Reviewer #4 (Remarks to the Author):

Overall, this is an interesting manuscript describing the role of transposable elements in tomato diversity. The authors have identified transposable element sequences in 600 varieties of tomato and shown that the TEs result in changes in gene expression phenotypic diversity. The authors show examples of TE-induced changes in fruit color and 2-phenylethanol levels. My main concern is the phenylethanol acyl transferase data. The only data shown are correlations between 2-phenylethanol and gene expression levels and presence or absence of the TE. It would greatly improve the manuscript if gene function was shown by in vitro enzyme assays or transgenic plants. In addition, it would be helpful to see that 2-phenethyl acetate levels are higher in varieties without the insertion. The data shown simply do not justify the conclusion that this is the causative gene.

What are the wild species mentioned in Figure 1 and line 48? Also, line 165 states “from wild tomato

relatives but present respectively at low and intermediate frequency in wild and SLC tomatoes" The difference between Solanum pimpinellifolium and other tomato relatives need to be made clear throughout the manuscript.
Figure 1C needs clarification.
What are the colors in Figure 1D? Do they match Figure 1A?
Figure 2C. Perhaps the color scale could be changed for the transposable elements with low frequency. As the figure is presented it is not useful to the reader. A separate scale for each TE would be much better.
Figure 2D legend. Length is spelled wrong.
Figure 3G: The pale blue and green colors are hard to distinguish.

We are grateful to the four reviewers for their comments, which greatly helped us to improve our manuscript.

Reviewers' comments:

Reviewer #1 (Remarks to the Author):

In the manuscript under consideration, the authors have presented their discovery of TEs/TIPs in tomato genome and their contributions to expression and phenotypic variance. I have significant concerns towards the validity of the GWAS and LD analysis.

The main concern is the quality of the input data of TE/TIPs. Unlike other analyses (such as evolution or impact to gene expression), GWAS replies on an accurate estimate of allele frequencies in the focal population. The repetitiveness in the TE related regions brings obstacles to such efforts. If we set the parameters to call TE/TIPs aggressively, it will bring false positives; however a rigorous filtering will lead to high false negatives, which hurt the analyses equality as false positives do.

In the current version of the manuscript, the FP and FN have not been thoroughly analyzed. Regarding FN, it is stated: "To limit false-negatives, non-carrier individuals with less than five reads negative coverage were considered as missing information or NA". This statement doesn't quantitatively show how large is the FN in the resulting dataset. For FP, it is stated: "we evaluated the presence of TIPs detected in the M82 cultivar on the high-quality assembled genome available for this accession. Consistent with the validation based on visual inspection, nearly 70% of TIPs detected in M82 were also found in the reference M82 genome, with COPIA insertions showing the highest specificity (77%)". I have two concerns of this statement. The percentage of 70% or 77% looks quite low for me: a 30% or 23% of FP will make the estimate of allele frequencies quite distorted for both GWAS and LD calculations, not to mention the unknown FN. Second, after reading the reference 42, that has generated the "high-quality assembled genome", I find that the TEs are not their focus, although it makes sense for me to use third-generation sequencing to assess TEs. We need more rigorous analyses of how is the quality of TE/TIPs reported by that paper.

We agree with the reviewer that the quality of the TIP calling is critical to derive meaningful allele frequencies. For the FP rate, we took two different approaches to estimate it as precisely as possible, and both approaches produced similar results (around 30% of FPs). We now provide a more detailed explanation of these two approaches (Lines 274-279). In addition we now mention that this estimated rate is similar to the one we obtained using the same pipeline for numerous *A. thaliana*

resequenced genome data and which we could validate experimentally using TE sequence capture (Baduel, Quadrana and Colot, Methods in Mol Biol, 2020 *in press*). We therefore feel confident about our estimated rate of FPs.
Unlike FPs, FNs cannot easily be detected as there is no TE annotation available (a complex task that is beyond the scope of the present study) for the well-assembled non-reference tomato genome recently produced, or a TE-sequence capture design like in *A. thaliana.* We can nonetheless assume that the rate of FNs should be similar in tomato and *A. thaliana*, where we determined it experimentally to be around 20% (Baduel, Quadrana and Colot, Methods in Mol Biol, 2020 *in press*). Furthermore, following the suggestion of Reviewer 3 (see below), we found that the rates of FP and FN obtained with our pipeline are similar to the ones recently reported in a comprehensive survey of multiple softwares specifically designed to detect TIPs using short-reads sequencing data (Vendrell-Mir et al. 2019).
As most TIPs are very rare variants, a FP rate of 30% will translate in most cases in calling a TIP in a single genome when there is none across all genomes examined. Similarly, a 20% FN rate should translate into rare TIPs being missed altogether, or more frequent TIPs being missed in one or very few genomes at most. As our GWA studies rely on variants with a frequency of at least 1% (i.e. detected in at least 6 of the 602 genomes examined), FPs and FNs can only have a minimal impact on the associations we detected and this impact will mostly diminish the power of our GWAS, leading to an underestimation of the contribution of TIPs to phenotypic variation. This point was made implicitly in the Discussion of the original version of the manuscript and we now make it explicit (Lines 216-218). Furthermore, we have now also performed empirical estimates of the specificity of our TIP-GWAS using permutation tests, which indicate that the likelihood of detecting false positive associations by chance is very low (from P=0.037 to P<0.001; Supplementary Fig 9 and Lines 353-358).

Because of the above limitation, the LD calculation between TEs and SNPs are also not convincing. Indeed, they found the LD between SNPs is higher than LD between TE and SNPs. This is actually consistent to my observations in other plant genomes, indicating high false negatives in the data related of structural variants. Assuming most SNPs and TEs are not functionally neural, I’d expect in general these two are similar (although some TEs or SNPs are in high LD with others because of selections). However the current short reads can’t offer good quality of calls in terms of both FP and FN. As such, the LD calculations may not support their claim regarding SNPs can’t tag the contributions of TEs towards the phenotypic variance.

Although we cannot exclude that some TIPs in low LD with nearby SNPs may be due to sensitivity and/or specificity issues, our additional analyses (suggested by Reviewer #2) indicate that low LD between TIPs and SNPs is largely explained by

TIPs being typically rarer in the population compared to SNPs (See our response to this point below and the revised manuscript Lines 120-124 and Supplementary Figure 2). Moreover, similar results were found in Arabidopsis, maize, grapevine and humans (Stuart et al. 2016 eLife; Yang et al. 2019 Nat. Genetics; Zhou et al. 2019 Nat. Plants and Sudmant et al. 2015 Nature), reinforcing the notion that most TIPs are in low LD with other variants.
We did not understand the comment “Assuming most SNPs and TEs being not functionally neural”, as most SNPs are neutral or mildly deleterious, in contrast to TIPs, which tend to be strongly deleterious.

To justify the validity of the work, the authors may conduct rigorous experimental validations to quantify the FP/FN of their TE/TIPs discoveries. Alternatively, a functional validation of the GWAS peaks may also serve as an evidence of the ultimate findings.

We feel that the additional analyses and explanations about FPs and FNs provided in the revised manuscript are sufficient to validate our work. Moreover, we include in the revised manuscript several new lines of evidence supporting our TIP-GWAS results. In addition to the TIP that was shown experimentally to cause variation in fruit color, we now describe a TIP identified through our GWAS and that was previously shown to cause variation in leaf morphology. Furthermore, using full-length cDNA Nanopore sequencing we provide strong molecular evidence that an intronic TIP associated with variation in 2-phenylethanol levels is causal.

Minor issues:

Page 18: The word ambiguous appears twice at line 393.
We have corrected this mistake.

Page 19: There is no citation regarding where they get the fruit color phenotype for 348 accessions. It might be helpful to provide more details on which seed banks databases they are using, what the raw data look like, and how they process the data.

We now provide a detailed description of the method used to compile phenotypic information of commercial cultivars by web data extraction (Lines 334-345). We have also included supplementary tables containing the raw data.

Page 20: Why only 10 permutations? This sounds a bit low for getting a random expectation.

We apologize for this error. The number of permutations performed was actually 10,000. We have corrected this in the new manuscript (Line 360).

As the minimal requirement in presentations of a GWAS signal, one may provide a full Manhattan plot (instead of only 1.2Mb in Chromosome 3 in your figure 3 or 2Mb in Chromosome 2 in your figure 5) to show the overall picture of the genetic architecture of the trait. Also, a QQ plot of expected and observed P-values needs to be included to justify that there is no inflation of the distribution of P-values.

We now provide full manhattan plots and qq-plots for the GWAS presented in Fig. 3 and 5.

Reviewer #2 (Remarks to the Author):

Review of “The impact of transposable elements on tomato diversity”

This manuscript examines transposable element insertion polymorphisms in previously sequenced tomato lines, examining several aspects of these polymorphisms. In brief, while some analyses presented here are interesting, the scholarship left a lot to be desired and too many results were un- or under-explored.

Regarding the scholarship, there are many papers that have examined TE polymorphisms previously, including in plants and including looking at associations with gene expression. These are far from being “ignored from most population genomic studies” (line 176). To name a few papers that examine TEs, these include in rice (Carpentier et al. 2019, Nature Communications), Arabidopsis (Stuart et al. 2016, eLife), Brachypodium (Eichten et al. 2016, Genome Research), carrot (Macko-Podgórni et al. 2019, Mobile DNA), Arabis (Rogivue et al. 2019, Molecular Ecology Resources), and multiple species of Capsella (Agren et al. 2016, Am J Bot; Uzunovic et al. 2019, Molecular Biology and Evolution). Though none of these are cited in the current submission, these are largely not obscure papers. In fact I can see that the current authors have cited some of these papers elsewhere, and for good reason: many of the same patterns they find in tomatoes are found in these species. For instance, in reviewing several of these studies, Gaut et al. (2018, Nature Plants) noted that “two-thirds of transposable elements (TEs) in a population survey of Arabidopsis thaliana were not in linkage disequilibrium with a nearby SNP”.

We thank the reviewer for raising his/her concern about this statement. However, we were careful to state that most population genomic studies have ignored TEs, as we are aware of the set of studies cited by reviewer 2. We now cite these studies in the Introduction (Line 35) and Discussion (Line 208). The point remains though that only a very small percentage of the tens of thousands GWAS performed to date have considered TIPs.

In addition, there are just as many similar studies in animal systems (especially Drosophila and humans) that deserve mention and that should be read for their careful consideration of many of the issues encountered here (e.g. Cridland et al. 2015, Genetics). Similarly, the idea that structural variants (including TEs) may help to improve GWAS and are often not tagged by SNPs has been a major focus of human studies for over a decade (Manolio et al. 2009, Nature).

We now cite and discuss these articles (Lines 90 - 91).

Overall, this manuscript would have been a lot better had it been placed in context within the field, exploring the patterns presented rather than applauding them.

Some specific comments about results presented in the manuscript:

-The authors report a different distribution of non-reference TE insertion polymorphisms relative to reference TEs. But I have to think that the way in which TEs were mapped to specific positions in the genome would ensure that this would always be the case. Because the tomato genome has large regions covered by TEs, the requirement for unique mapping of TE polymorphisms both upstream and downstream (lines 368-371) would automatically exclude them from being mapped to highly repetitive regions. Exploring the "mappability" of TEs to specific parts of the genome would help to put these distributions in context. Additionally asking whether specific types of genes were in regions that were more mappable than others would also help to make specific GO-term overrepresentation more convincing as a biological pattern.

Our results indicate that different TE families display contrasted TIP landscapes, with *COPIA* and *GYPSY* TIPs preferentially located within or far away from genes, respectively, and with *COPIA* TIPs affecting specific GO terms. These results are not consistent with mappability bias as all families should be affected similarly. Nonetheless, we have determined short-read mappability genome-wide, which, as expected, is reduced within pericentromeric regions. We have included this additional analysis in Fig. 2a,2b and 2c. These new series of experiments further support our initial observations.

-Lines 88-90: "TIPs associated with two-fold or more changes in gene expression are proportionally more frequent when located in exons and introns (43% and 37%, respectively) than in other gene compartments (Fig. 2d)." I actually don't see this in the figure. The light blue and dark blue bars seem to be the same height in all compartments.

Each stacked bar represents the total fraction of genes with expression changes greater than 2-fold (i.e. one log2 change) and the different bin colors represent changes of different magnitude. Thus, greater height of the bars for exons and introns indicate that TIPs associated with changes greater than two-fold in gene expression are more frequently located in these two genic compartments. We have clarified the figure and explained the binning procedure in the methods section (Lines 324-326).

-Lines 114-115: Are there fewer TEs in high LD with SNPs because of their lower average population frequency? If you frequency-match TEs and SNPs, is there still lower LD? Why would this be the case?

We have included a new analysis showing that TIPs with lower LD with nearby SNPs tend to be rarer than those with intermediate LD or high LD (Supplementary Figure 2). These results are consistent with observations in A. thaliana (Stuart et al 2016, eLife), maize (Yang et al 2019, Nat. Genet) and grapevine (Zhou et al. 2019 Nat. Plants), which we now also discuss in the manuscript (Lines 120-121).

Reviewer #3 (Remarks to the Author):

The manuscript submitted by Dominguez et al. reports the identification of transposable elements insertion polymorphisms in tomato and a GWAS analysis to tentatively identify association between some TIPs and agronomic traits in this crop (fruit quality traits). The paper is well written, the study is of interest to plant genomicists and the functional impact of structural variations in plants is definitely a trendy topic. I have, however, several major concerns about this manuscript the way it stands :

1 – The functional relationships between TIPs and fruit quality (whether identified using a candidate gene approach or through GWAS) are tentative. The authors do not provide any functional validation of what they claim and no causal relationship is secured for any of the traits (except for the association of a TIP with fruit colour, used here as positive control). Therefore, most of the results presented by the authors are over-interpreted and the result section should be rewritten. In

particular, the claims made in paragraph p6, l01-110 are pure speculation. This should be removed or rephrased.

We agree that GWAS results cannot prove causality per se and we have carefully edited the manuscript to avoid claiming otherwise.

2 – The authors have used a « refined version of SPLITREADER » . What does this exactly mean ? Why did they have to refine their existing software ? Was it because it was originally designed for a 100Mbp genome and did not work for the much larger genome of tomato ? If yes it should be mentioned. What was changed (« refined ») ? In addition, the authors have completely ignored the existing literature on the subject of TIPs detection from NGS data. This should be corrected. For instance Vendrell-Mir et al. (Mob. DNA 2019) have conducted a major comparative analysis of existing softwares. How does the « refined » version of SPLIREADER compare to these ? In addition, a similar survey was conducted on rice (Carpentier et al., 2019). This work is not cited, which considerably weakens the present manuscript.

We have expanded the methods section to describe in greater detail the new version of SPLITREADER ands to provide additional measurements of the sensitivity and specificity of the pipeline. In addition, we have discussed the manuscript of Carpentier et al 2019 in the context of the study of TIPs in crops (Line 208) and Vendrell-Mir et al. 2019 in the context of specificity and sensitivity of TIPs detection (Lines 278-279).

3 – This work is not the first report of the use of TIPs-GWAS. It was reported on Brassica napus (Song et al., Nature Plant, 2020) and rice (Fuentes et al., Genome Research 2019 ; Akakpo et al. , New phytologist 2020). Again, the authors should have acknowledged it and compared and discussed their results with the previously published ones on two other crops.

We apologize for this oversight and now cite the three articles (Line 35).

Reviewer #4 (Remarks to the Author):

Overall, this is an interesting manuscript describing the role of transposable elements in tomato diversity. The authors have identified transposable element sequences in 600 varieties of tomato and shown that the TEs result in changes in gene expression phenotypic diversity. The authors show examples of TE-induced changes in fruit color and 2-phenylethanol levels. My main concern is the phenylethanol acyl transferase data. The only data shown are correlations between 2-phenylethanol and gene expression levels and presence or absence of the TE. It

would greatly improve the manuscript if gene function was shown by in vitro enzyme assays or transgenic plants. In addition, it would be helpful to see that 2-phenethyl acetate levels are higher in varieties without the insertion. The data shown simply do not justify the conclusion that this is the causative gene.

We agree that GWAS results are correlative. However, our work demonstrates that the use of TIPs can greatly improve our ability to link genetic variation to trait variation and we provide in the revised manuscript one additional example of causality, which concerns leaf morphology (Lines 127-130). Furthermore, we have expanded further the analysis of *Solyc02g079490* in the revised manuscript. Notably, we have performed full-length cDNA Nanopore sequencing to determine how the *COPIA* LTR-retrotransposon insertion affects this gene*.* Our results indicate that the insertion likely generated an hypomorphic allele that produces multiple non-functional isoforms, thus providing a plausible explanation for the accumulation of 2-phenylethanol in accessions that carry it (Lines 178-198). However, a full characterization of *Solyc02g079490,* including through genome editing, is beyond the scope of our study.

What are the wild species mentioned in Figure 1 and line 48? Also, line 165 states "from wild tomato relatives but present respectively at low and intermediate frequency in wild and SLC tomatoes" The difference between Solanum pimpinellifolium and other tomato relatives need to be made clear throughout the manuscript.

We have now included in the Source Data File a table with a detailed description of each accession. We have revisited to the best of our abilities the whole manuscript to make clear the distinction between tomato groups.

Figure 1C needs clarification.
We have modified the axes and legend of Figure 1C.

What are the colors in Figure 1D? Do they match Figure 1A?
Yes, colors in Fig. 1D match those of 1A. We have modified the figure and legend to facilitate comprehension.

Figure 2C. Perhaps the color scale could be changed for the transposable elements with low frequency. As the figure is presented it is not useful to the reader. A separate scale for each TE would be much better.

We do not understand this comment. TIPs are also relatively frequent over genes for the MuDR, hAT and CACTA superfamilies, yet no enrichment is detected for any GO term.

Figure 2D legend. Length is spelled wrong.
This is now corrected.

Figure 3G: The pale blue and green colors are hard to distinguish.
We have changed the color code to facilitate the readability of the figure.

Reviewer #1 (Remarks to the Author):

In the revised manuscript, the authors have conducted some work to address my comments. However, I still have substantial concerns on the validity of using the current dataset to carry out association mapping.

For the first concern, FP/FN, the authors have agreed that the FP and FN are very high, i.e., 30% and 20% respectively; however they argued that this will only cause underpowered association study, leading to the claim that the main finding remains correct. I am afraid that I cannot agree with this argument. In the practice of GWAS, if a SNP has more than 20% missing calls, it will be removed from the analyses. With this in mind, 30% of FP and 20% of FN are simply too high. (In fact, given the fact that most tools for TE discovery use conservative cutoffs to avoid FP and hence leave higher FN, I am afraid the actual FN may be higher than 20%.) Indeed, in the QQ-plot (Fig 5a), it is clear that the type-I error is under control for SNPs associations however not for TIPs. As these two distributions are calculated for the same phenotype and the same population structure, this QQ-plot does indicate a high false discovery rate in the TIP association mapping.

For my second concern on LD, I agree with the authors' explanations. (Sorry for my typo in the original review comments!) However, the evidence brought by the authors, the MAF of TEs being low, highlights the substantial impact of FPs in GWAS. For a TE/TIP with a moderate MAF, say 0.1 or 0.2, a FP of 30% will generate a huge potential of false associations.

Nevertheless, despite my reservation of the statistical association, I appreciate that the authors did conduct experiments to validate their key discoveries. If I understand the revised manuscript correctly, there is no sequencing experiment using PCR or long-reads to validate that the MAF of causal TIPs are indeed correctly assessed. Instead there are two lines of evidence to show that the function of the variant is causal to the phenotype. I am a statistician without solid background to interpret such functional experiments; therefore have to leave this question to other reviewers and the editor to decide.

Reviewer #2 (Remarks to the Author):

The authors have adequately addressed my comments. While I don't think that minor differences in the distribution of two TE families constitutes good evidence for a lack of detection bias, they have at least acknowledged this limitation.

My only remaining comment is: if TEs with lower LD have lower allele frequencies, then this is not surprising at all. The same would be true of SNPs at lower frequencies. So I still can't tell whether TEs are special, or whether the authors have simply added a bunch of markers to a GWAS.

Reviewer #3 (Remarks to the Author):

THe authors have made some progress in revising their manuscript. Previous reports have been cited, but none of them have been discussed !!! What did Carpentier et al. conclude from their work on rice ? What did Fuentes et al. and Akakpo et al. find in their GWAS analyses ? what did Zhou et al. find in grapevine ? What did Macko-Podgorni find in carrots. THe reader should find in the present manuscript a thorough examination of these earlier reports which should enrich the discussion of this work on tomato.
Some of the earlier discoveries are similar to what is observed in this manuscript in tomato. This should be discussed. Isn't it what science is about ?

I therefore consider that the manuscript is not suitable for publication in the present state.

Reviewer #4 (Remarks to the Author):

The authors have thoughtfully responded to the reviewers' comments. Although they did not show the function of PPEAT, they did show that RNA isoforms resulting in different proteins, when the TE was inserted in an intron. They have also stated that the function is putative. I think it would be good to remove Figure 5D, since this function is not proven. It is enough to state in the text that this is the possible function of the protein.

Reviewer #1:

In the revised manuscript, the authors have conducted some work to address my comments. However, I still have substantial concerns on the validity of using the current dataset to carry out association mapping.

We would like to thank reviewer #1 for his assessments and comments, which we have answered below. We believe that as a result of the additional analyses we provide, the revised manuscript is substantially improved.

For the first concern, FP/FN, the authors have agreed that the FP and FN are very high, i.e., 30% and 20% respectively; however they argued that this will only cause underpowered association study, leading to the claim that the main finding remains correct. I am afraid that I cannot agree with this argument. In the practice of GWAS, if a SNP has more than 20% missing calls, it will be removed from the analyses. With this in mind, 30% of FP and 20% of FN are simply too high. (In fact, given the fact that most tools for TE discovery use conservative cutoffs to avoid FP and hence leave higher FN, I am afraid the actual FN may be higher than 20%.) Indeed, in the QQ-plot (Fig 5a), it is clear that the type-I error is under control for SNPs associations however not for TIPs. As these two distributions are calculated for the same phenotype and the same population structure, this QQ-plot does indicate a high false discovery rate in the TIP association mapping.

We agree that in addition to reducing sensitivity, stochastic noise can result in false associations in GWAS. To address further this issue, we have implemented a series of additional filters to obtain robust associations. In particular, and as suggested by reviewer #1, we have excluded all TIPs with more than 20% of missing data (Lines 130-131 and 363 of the revised manuscript), which reduced the number of detected associations to 53 from 179 initially. Given that low quality calls can increase the probability of finding false associations, we also inspected visually all of these 53 TIP associations and we removed or corrected false positive and negative TIPs, respectively. This step (described in Lines 134-135, 160-161 and 372-375 of the revised manuscript) produced a final set of 40 highly robust TIP associations (9 for morphological traits and 31 for metabolic traits), which are the only ones we now consider. Importantly, this robust set includes the TIPs at *PSY1* and *PPEAT* that are associated with fruit color and tomato flavor, respectively. Furthermore, it also contains the TIP at *BLI2* associated with leaf morphology and the association is now much stronger for the TIP than for any SNPs (Figure 3b,c), suggesting that the TIP is the causal variant in this case too. Finally, the observation that TIPs have a much larger effect size than SNPs and are preferentially associated with volatiles still holds true (Figure 4b,c), which confirms that our robust set of TIP associations are not spurious. Indeed, this type of metabolites are implicated in defense response and/or interaction with other organisms and are controlled by genes that are recurrently targeted by TEs (Fig. 2c).

For my second concern on LD, I agree with the authors' explanations. (Sorry for my typo in the original review comments!) However, the evidence brought by the authors, the MAF of TEs

being low, highlights the substantial impact of FPs in GWAS. For a TE/TIP with a moderate MAF, say 0.1 or 0.2, a FP of 30% will generate a huge potential of false associations.

To assess the FP rate more precisely, we considered a random set of 38 TE insertions with no detectable associations but whose presence was confirmed visually in at least one genome. For each of these 38 TE insertions, as well as for the 18 TE insertions with robust associations analyzed above, we could not detect across 516 accessions any false positive by visual inspection (i.e. 28,896 visual inspections in total) of short-reads alignements to the tomato reference genome sequence. Thus, we can conclude that the false positive rate is negligible once a TE insertion has been confirmed visually in at least one genome (Lines 278-281). Moreover, using these 56 curated TE insertions we confirmed the lower LD observed for TIPs compared to SNPs (Lines 121-123).

Nevertheless, despite my reservation of the statistical association, I appreciate that the authors did conduct experiments to validate their key discoveries. If I understand the revised manuscript correctly, there is no sequencing experiment using PCR or long-reads to validate that the MAF of causal TIPs are indeed correctly assessed. Instead there are two lines of evidence to show that the function of the variant is causal to the phenotype. I am a statistician without solid background to interpret such functional experiments; therefore have to leave this question to other reviewers and the editor to decide.

We thank Reviewer #1 for his/her comment and for acknowledging that the additional experiments presented in the new version of the manuscript validated our key discoveries. As mentioned before, all associated TIPs have now been validated visually. In addition, we have also performed PCR-based genotyping of the TIPs associated with fruit color and 2-phenylethanol and the presence/absence of TE insertions were confirmed in each case (Supplementary Figure 7).

Reviewer #2:

The authors have adequately addressed my comments. While I don't think that minor differences in the distribution of two TE families constitutes good evidence for a lack of detection bias, they have at least acknowledged this limitation.

We thank Reviewer #2 for his/her appreciation of our revised manuscript.

My only remaining comment is: if TEs with lower LD have lower allele frequencies, then this is not surprising at all. The same would be true of SNPs at lower frequencies. So I still can't tell whether TEs are special, or whether the authors have simply added a bunch of markers to a GWAS.

Our analyses clearly show that because TEs typically create major effect alleles upon integration, transposition constitutes a sui generis source of variation. This special contribution of TIPs is further accentuated by the observation that TEs have strong integration preferences.

Reviewer #3:

The authors have made some progress in revising their manuscript. Previous reports have been cited, but none of them have been discussed !!! What did Carpentier et al. conclude from their work on rice ? What did Fuentes et al. and Akakpo et al. find in their GWAS analyses ? what did Zhou et al. find in grapevine ? What did Macko-Podgorni find in carrots. THe reader should find in the present manuscript a thorough examination of these earlier reports which should enrich the discussion of this work on tomato.

Some of the earlier discoveries are similar to what is observed in this manuscript in tomato. This should be discussed. Isn't it what science is about ?

I therefore consider that the manuscript is not suitable for publication in the present state.

We are very sorry about the frustration expressed by reviewer #3 as we believe that we provided a fair response to the third point raised during his/her initial review. Indeed, our findings were placed in the framework of previous studies in the following sentence of Discussion:

“In this context, we show that TIPs, which to date have been ignored from population genomic studies in tomato, are an important diversifying force to consider, as has been proposed for other plant species 27,33,34,52–57.”

Nonetheless, we have now modified (italics) the following sentences to relate explicitly our study in tomato to previous studies in other plant species.

Lines 213-216 *“...For instance, GWAS in rice for grain length and width using structural variants or TIPs, respectively, uncovered associations that could not be detected using SNP data, which in the case of grain width can be attributed to the associated TIPs being very rare and in low LD with nearby SNPs (Fuentes et al. 2019; Akakpo et al. 2020).*

Lines 217-220 *“... our findings reinforce the notions that TIPs and SNPs contribute distinct phenotypic variants and that TIPs identified in GWAS as leading variants are likely causal (Fuentes et al. 2019; Akakpo et al. 2020; Song et al. 2020; Zhou et al. 2019), which open up the way for their use for future breeding*.

Lines *221-225 “We based our study on TIPs using 467 TE families that represent most of the retrotransposons and DNA transposons present in the reference tomato genome. However, we did not consider small-length TE families, such as SINEs and MITEs, which are also likely to contribute to phenotypic diversity (Macko-Podgorni et al 2019) but that are difficult to analyze using short-reads because of their small size and very high copy number in many genomes. ”*

Lines 227-229 *"...studies in A. thaliana, tomato, maize and grapevine based on long-read sequencing technologies revealed thousands of structural variants, most of which involve TEs and other repeats (Yang et al. 2019; Alonge et al. 2019; Michael et al. 2017; Zhou et al. 2019)"*

Reviewer #4:

The authors have thoughtfully responded to the reviewers' comments. Although they did not show the function of PPEAT, they did show that RNA isoforms resulting in different proteins, when the TE was inserted in an intron. They have also stated that the function is putative. I think it would be good to remove Figure 5D, since this function is not proven. It is enough to state in the text that this is the possible function of the protein.

We thank Reviewer #4 for his/her appreciation of our revised manuscript and we have followed his/her recommendation to remove Figure 5D.

REVIEWERS' COMMENTS:

Reviewer #1 (Remarks to the Author):

In the revised manuscript, the authors have conducted further filtering to improve the average quality of the TIP calls. Additionally, they have carried out PCR experiments together with manual inspections to ensure the main discoveries are solid. As it is, I am more comfortable on the reported main discoveries.

Although in general I feel it is not appropriate to conduct GWAS in repetitive regions based on NGS-assessed questionable calls, I am OK to accept this work for publication if the editor and other reviewers strongly support this.

Reviewer #1 (Remarks to the Author):

In the revised manuscript, the authors have conducted further filtering to improve the average quality of the TIP calls. Additionally, they have carried out PCR experiments together with manual inspections to ensure the main discoveries are solid. As it is, I am more comfortable on the reported main discoveries.

We are glad to see that our additional analyses provide more solidity to our results and that reviewer #1 is now more comfortable with the reported discoveries.

Although in general I feel it is not appropriate to conduct GWAS in repetitive regions based on NGS-assessed questionable calls, I am OK to accept this work for publication if the editor and other reviewers strongly support this.

Thanks to the stringent validations implemented following previous suggestions of reviewer #1, we now report only robust associations with TIPs. We are happy to see that reviewer #1 accept the publication of our work in conformity with the editor and the other three reviewers.
